# Characterization of subcellular localization of eukaryotic clamp loader/unloader and its regulatory mechanism

**DOI:** 10.1038/s41598-021-01336-w

**Published:** 2021-11-08

**Authors:** Su Hyung Park, Seong-jung Kim, Kyungjae Myung, Kyoo-young Lee

**Affiliations:** 1 Center for Genomic Integrity, Institute for Basic Science, Ulsan, 44919 Korea; 2 Department of Biological Sciences, Ulsan National Institute of Science and Technology, Ulsan, 44919 Korea; 3 Department of Biomedical Engineering, Ulsan National Institute of Science and Technology, Ulsan, 44919 Korea

**Keywords:** Nucleoskeleton, Replisome

## Abstract

Proliferating cell nuclear antigen (PCNA) plays a critical role as a processivity clamp for eukaryotic DNA polymerases and a binding platform for many DNA replication and repair proteins. The enzymatic activities of PCNA loading and unloading have been studied extensively in vitro. However, the subcellular locations of PCNA loaders, replication complex C (RFC) and CTF18-RFC-like-complex (RLC), and PCNA unloader ATAD5-RLC remain elusive, and the role of their subunits RFC2-5 is unknown. Here we used protein fractionation to determine the subcellular localization of RFC and RLCs and affinity purification to find molecular requirements for the newly defined location. All RFC/RLC proteins were detected in the nuclease-resistant pellet fraction. RFC1 and ATAD5 were not detected in the non-ionic detergent-soluble and nuclease-susceptible chromatin fractions, independent of cell cycle or exogenous DNA damage. We found that small RFC proteins contribute to maintaining protein levels of the RFC/RLCs. RFC1, ATAD5, and RFC4 co-immunoprecipitated with lamina-associated polypeptide 2 (LAP2) α which regulates intranuclear lamin A/C. *LAP2α* knockout consistently reduced detection of RFC/RLCs in the pellet fraction, while marginally affecting total protein levels. Our findings strongly suggest that PCNA-mediated DNA transaction occurs through regulatory machinery associated with nuclear structures, such as the nuclear matrix.

## Introduction

The nuclear lamina, located just beneath the inner nuclear membrane (INM), is composed of A- or B-type lamins and their many lamina-associated proteins^1,2^. The A-type lamins (lamin A and C, lamin A/C) and B-type lamins, in a complex with lamina-associated polypeptide (LAP)-Emerin-MAN1 (LEM) domain proteins and lamin B receptor of the INM, respectively, tether heterochromatic genomic regions to the nuclear periphery^3^. These structures shape the spatial architecture of chromatin, which is closely related to gene regulation and replication timing in the interphase nucleus^1,4,5^.

Unlike B-type lamins, which are primarily present in the nuclear lamina, about 10% of lamin A/C are also found in the nucleoplasm^6,7^. In the nucleoplasm, lamin A/C binds to euchromatin, which is regulated by association with the nucleoplasmic LEM domain protein LAP2α^1^. Nucleoplasmic lamin A/C limits free diffusion of fluorescently tagged genomic regions but depletion of LAP2α does not affect chromatin diffusion^8^. However, another recent report showed that depletion of LAP2α reduces the mobility of nucleoplasmic lamin A/C, which in turn reduces chromatin diffusion^9^. This suggests that nucleoplasmic lamins, like the peripheral lamina, contribute to chromatin architecture. However, it is still not clear whether internal lamins also provide structural platforms for specific functions, such as gene regulation and DNA replication and repair.

During the S phase in eukaryotic cells, the entire genome is replicated in units of replication domains^5,10^. Labeling of replicating DNA with nucleotide analog bromodeoxyuridne (BrdU) shows a distinctive focal pattern where each focus represents an active replication domain^11,12^. In early S phase, foci appear throughout the entire nucleoplasm, whereas foci in mid- and late S phase appear only in the nuclear periphery and outside the nucleoli. In a single replication domain, three to five out of many licensed origins fire simultaneously and initiate replication at a specific location called the replication factory^13^. In the replication factory, replication proteins associated with replication forks from the origin firing and timely recruited to replication forks, participate in DNA synthesis, Okazaki fragment maturation, sister chromatid cohesion, DNA repair, and checkpoint activation.

Proliferating cell nuclear antigen (PCNA), the eukaryotic sliding clamp, plays a critical role as a processivity factor for replicative DNA polymerases and as a binding platform for many proteins involved in different DNA transactions^14^. The replication factor C (RFC) complex, a pentameric AAA + ATPase complex composed of a large subunit, RFC1, and four small RFC proteins, RFC2, 3, 4, and 5^15,16^, loads PCNA onto DNA at the double-strand/single-strand DNA junction with the 3′-hydroxyl group. Eukaryotes have three RFC-like complexes (RLCs), which have overlapping, as well as distinct, cellular tasks. Each RLC complex is composed of one of three large subunits: CTF18, ATAD5 (Elg1 in yeast), or RAD17 (Rad24 in budding yeast) and four small subunits of RFC2-5^17^. The CTF18-RLC complex loads PCNA in a manner similar to the RFC complex, but unlike RFC-loaded PCNA, CTF18-RLC-loaded PCNA participates in cohesion establishment and checkpoint activation^18^. After DNA synthesis is complete, ATAD5-RLC unloads PCNA from DNA^19–21^. RAD17-RLC loads another ring-shaped clamp, RAD9-RAD1-HUS1 (the 9-1-1 complex, Ddc1-Rad17‐Mec3, in budding yeast)^22^, which plays a role in checkpoint activation^23,24^.

Previous reports show that replication along each replication domain occurs in a spatially stationary or minimally mobile fashion in living cells^25,26^. Several elements support this stationary replication. Chromatin-intrinsic features help maintain stationary replication at the level of the replication domain. About 40% of the genome is bound to the lamina through lamina-associated domains on chromatin^27^. In addition, chromatin is self-organized in a megabase-sized topologically associating domain (TAD)^28,29^. Each TAD serves as a structural platform for contacts between promoters and enhancers, and at least one TAD may correspond to a replication domain^30,31^. It has been suggested that adjacent TADs are linked by interaction between genome-dispersed repetitive sequences^31^. Associations of chromatin with lamina and interactions between TADs stabilize chromatin architecture, which can contribute to the relatively stationary replication.

Replication machineries may also be tethered to immobile support in the nuclear interior, further contributing to stationary replication^32^. PCNA and lamin A/C directly interact, and replicative DNA polymerases associate with lamin A/C^33–35^. In addition, a study in budding yeast shows that DNA is replicated by passing through a stationary replication factory^36^. Due to the multiple essential roles of PCNA in cellular processes, PCNA loading and unloading needs to be accurately regulated. However, it has not been investigated how the PCNA loading and unloading complexes perform their tasks within the chromatin architecture and whether they are tethered to a mechanical support for stationary replication. To answer these questions, we analyzed the structural localization of RFC/RLCs and other replication-related proteins. We discovered that all RFC/RLC proteins were found in nuclease-resistant protein fractions; this localization was not affected by cell cycle or DNA damage. We also identified the protein–protein interaction between the RFC/RLC proteins and LAP2 proteins and found reduced association of RFC/RLC proteins with nuclease-resistant structures in mouse *Lap2α* knockout cells.

## Results

### RFC/RLC proteins are present in nuclease- and high salt-resistant protein fractions

**Figure 1:**
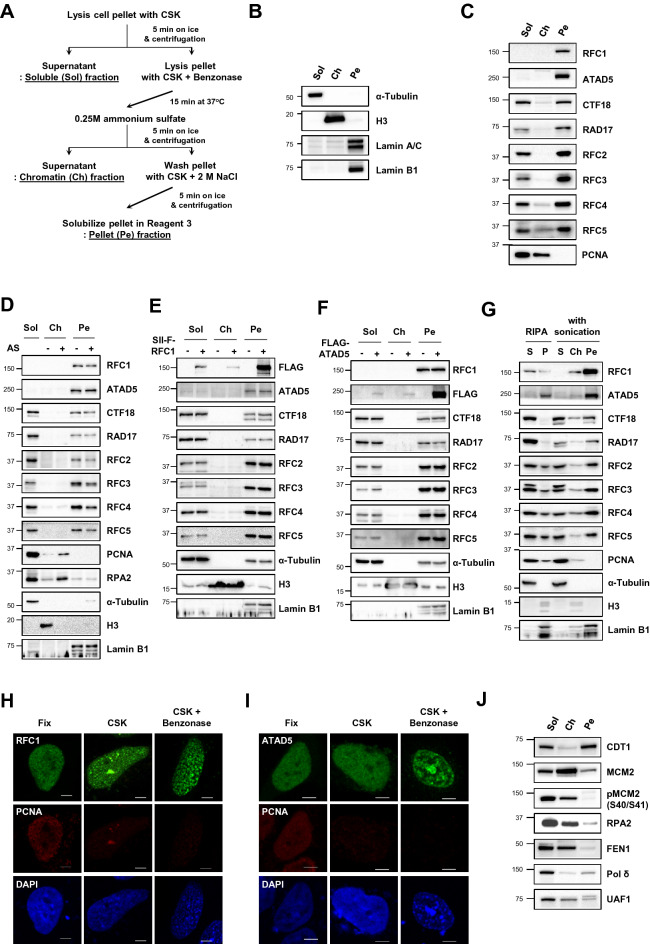
RFC/RLC proteins are present in nuclease- and high salt-resistant protein fraction. (**A**) The scheme for protein fractionation method. Detailed procedure was described in the method section. (**B**–**F**, **J**) HeLa cells were fractionated as the scheme in (**A**). Soluble (Sol), chromatin (Ch) and pellet (Pe) fractions were subjected to immunoblotting as indicated. (**D**) Chromatin and pellet fractions were prepared with or without ammonium sulfate (AS) step (−). (**E**, **F**) HeLa cells were transfected with Strep-tag II-FLAG-tagged RFC1 (SII-F-RFC1) (E) or 3xFLAG-tagged ATAD5 (FLAG-ATAD5) cDNA 48 h before protein fractionation. (**G**) (*left two lanes*) HeLa cells were lysed with RIPA buffer without Benzonase nuclease treatment, and soluble (S) and pellet (P) proteins were subjected to immunoblotting. (*right three lanes*) HeLa cells were fractionated as the scheme in (**A**) but sonication step was added after Benzonase nuclease treatment. (**H**, **I**) HeLa cells were transfected with mNeonGreen-tagged RFC1 (**H**) or mNeonGreen-tagged ATAD5 (**I**) cDNA. Forty-eight hours after transfection, cells were fixed or pre-treated with CSK alone or CSK plus Benzonase with the same condition as in (**A**) and then fixed for immunostaining. Scale bar = 5 μm. (**B**–**G**, **J**) Uncropped blot images are presented in Supplementary Fig. 5.

To investigate the structural localization of RFC/RLCs and other replication-related proteins, we sequentially extracted proteins from asynchronous human HeLa and HEK293T cells (Fig. 1A). We chose a nonionic detergent-based extraction method to preserve protein–protein interactions during subcellular fractionation^37^. First, we isolated cytoplasmic and nucleoplasmic proteins using cytoskeleton (CSK) buffer (soluble fraction). Next, we isolated nuclease-susceptible chromatin-associated proteins by digesting the pellet with nuclease (chromatin fraction). After washing the pellet with 2 M NaCl, we solubilized nuclease- and high salt-resistant nuclear matrix (NM) proteins and proteins associated with the NM or nuclease-resistant chromatin (pellet fraction). We confirmed the feasibility of the fractionation procedure by the detection of α-tubulin, histone H3, and lamin proteins in the expected fraction, respectively (Fig. 1B and Supplementary Fig. 1).

We next examined the fractionation profile of the RFC/RLC proteins (Fig. 1C and Supplementary Fig. 1). As expected, we detected PCNA in both soluble and chromatin fractions. Conversely, RFC/RLC proteins were present in the soluble and pellet fractions but not in the chromatin fraction. In particular, RFC1 and ATAD5 were detected only in the pellet fraction. Considering the roles of the RFC and ATAD5-RLC in PCNA loading/unloading, this profile was unexpected. Therefore, we tested the possibility that this profile was a result of our fractionation procedure. First, we examined the effects of ammonium sulfate, which is commonly used to precipitate proteins in various purification assays^38^. We considered the possibility that the 3% ammonium sulfate solution used to elute chromatin, as well as chromatin-associated proteins such as PCNA and RPA2 (Fig. 1D), could unintentionally precipitate RFC/RLC proteins as well. However, even when we omitted the ammonium sulfate step from the fractionation procedure, we did not detect RFC/RLC proteins in the soluble and chromatin fractions (Fig. 1D).

It is also possible that RFC1 and ATAD5 proteins are intrinsically unstable and form aggregates during the fractionation procedure, leading to detection only in the pellet. When we overexpressed Strep-tag II-FLAG-tagged RFC1 and 3xFLAG-tagged ATAD5 in the cells, we did detect both proteins in the soluble and chromatin fractions, albeit at much lower levels than in the pellet fraction (Fig. 1E,F). In addition, when we used Radioimmunoprecipitation assay (RIPA) buffer containing the ionic detergents sodium dodecyl sulfate and sodium deoxycholate, which potentially denature proteins or disrupt protein–protein interaction, a significant amount of RFC1 was solubilized and nearly all CTF18 and RAD17 proteins were detected in the RIPA-soluble fraction without nuclease treatment or sonication (Fig. 1G). However, ATAD5 was still detected only in the RIPA-insoluble pellet with a portion of small RFC proteins (Fig. 1G). ATAD5 was partly solubilized in the RIPA buffer and much milder buffer X upon nuclease treatment followed by sonication^39^. Accordingly, when we added the sonication step after nuclease treatment, we detected only small amounts of RFC1 and ATAD5 in the chromatin fraction, while most RFC1 and ATAD5 remained in the pellet fraction (Fig. 1G). These results minimize the possibility that RFC/RLC proteins are inherently vulnerable to our fractionation procedure and suggest that ATAD5-RLC proteins are more resistant to harsh extraction techniques compared to other RFC/RLC proteins. Finally, when we expressed mNeonGreen fluorescence protein-tagged RFC1 or ATAD5 in cells, we detected the mNeonGreen signal in the nucleus even after extracting soluble and chromatin-associated proteins using CSK and nuclease treatment as exemplified by the step-wise disappearance of PCNA signals (Fig. 1H,I). Interestingly, most of the mNeonGreen-RFC1 and mNeonGreen-ATAD5 signals overlapped with the DAPI signals remained after CSK and nuclease treatment. Collectively, our data suggest that a majority of RFC and ATAD5-RLC proteins are associated with the chromatin or NM inaccessible to exogenous nucleases.

We next examined the fractionation profile of several replication-related proteins. Origin licensing occurs during early G1 phase, beginning with origin recognition by the origin recognition complex and followed by chromatin licensing and DNA replication factor 1 (CDT1)-mediated minichromosome maintenance (MCM) helicase loading^40,41^. CDT1 was primarily detected in the soluble and pellet fractions, while MCM helicase subunit MCM2 was detected predominantly in the chromatin fraction and less abundantly in the pellet fraction (Fig. 1J and Supplementary Fig. 1). The phosphorylation of MCM2 at serine 40 and 41 residues, mediated by cell division cycle 7 (CDC7) kinase and cyclin-dependent kinase 2 (CDK2), respectively, is associated with replication initiation^42^. MCM2 phosphorylation at serine 40 or 41 was not detected in the pellet fraction (Fig. 1J). This result is in line with a previous report that showed that MCM2 is tightly associated with chromatin but not with the NM upon replication initiation^43^. Replication protein A 2 (RPA2), a single-stranded DNA binding protein, and PCNA-interacting flap endonuclease 1 (FEN1), an Okazaki fragment processing enzyme, had a fractionation profile similar to that of PCNA (Fig. 1J and Supplementary Fig. 1). However, DNA polymerase δ, a replicative polymerase for the lagging strand which also interacts with PCNA, was detected more highly in the pellet than in the chromatin fraction. These results suggest that replication-related proteins that commonly require PCNA binding perform their tasks with a different subcellular localization profile.

### The fractionation profile of RFC/RLC proteins is maintained throughout cell cycle with distinct mitotic phosphorylation of large subunits

**Figure 2:**
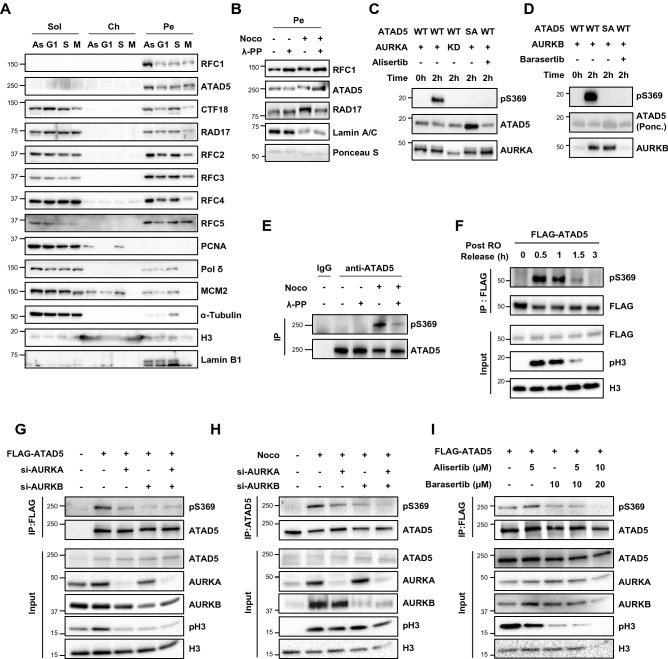
The fractionation profile of RFC/RLC proteins is maintained throughout cell cycle. (**A**) HeLa cells were arrested at the mitotic (M) phase using thymidine-nocodazole block and the shaked-off cells were re-seeded in fresh media and incubated for 4 h (G1) and 12 h (S). Cells at each phase and asynchronous (As) cells were collected, fractionated and subjected to immunoblotting. (**B**) HeLa cells asynchronous or arrested at the mitotic phase by treatment of nocodazole (Noco) were fractionated. Lambda phosphatase (λ-PP) (400 Unit/sample) was added during the nuclease treatment step and pellet (Pe) fraction was subjected to immunoblotting. (**C**, **D**) The in vitro kinase assay. The purified wild-type (WT) or S369A (SA) mutant of ATAD5 fragment (residues 1–400) was incubated with the recombinant wild-type (+) or kinase-dead (KD) AURKA (**C**) or AURKB (**D**) for indicated times. Alisertib (**C**) or Barasertib (**D**) was added in the reaction as indicated. The reaction samples were subjected to immunoblotting. (**D**) ATAD5 was detected by Ponceau S (Ponc.) staining. (**E**) Whole-cell extracts were prepared from HEK293T cells asynchronous (Asy) or arrested at the mitotic phase by treatment of nocodazole, and then subjected to immunoprecipitation with anti-ATAD5 antibody. Lambda phosphatase (λ-PP) (400 Unit/sample) was added during the nuclease treatment step. (**F**) HEK293T cells were transfected with 3xFLAG-tagged ATAD5 fragment (residues 1–400) cDNA. One day after, cells were arrested at the G2 phase by treatment of RO-3306 (RO) for 16 h and released from the arrest during the indicated time. Whole-cell extracts were prepared for immunoprecipitation with anti-FLAG antibody. (**G**–**I**) HEK293T cells were transfected with a combination of 3xFLAG-tagged ATAD5 cDNA and *AURK* siRNAs (**G**), *AURK* siRNAs (**H**), or 3xFLAG-tagged ATAD5 cDNA (**I**) as indicated. After transfection, cells were arrested at the mitotic phase by treatment of nocodazole. (**I**) Cells were treated with kinase inhibitors. Then whole-cell extracts were prepared for immunoprecipitation with anti-ATAD5 (**H**) or anti-FLAG antibody (**G**, **I**). (**A**–**I**) Uncropped blot images are presented in Supplementary Fig. 5.

PCNA loading and unloading primarily occurs during S-phase DNA replication. Therefore, it is expected that a portion of RFC, CTF18-RLC, and ATAD5-RLC would be associated with chromatin during PCNA loading and unloading during S phase. However, since RFC1 and ATAD5 were detected only in the pellet and not in the chromatin fraction (Fig. 1C), we examined whether the fractionation profile of RFC/RLC proteins changed at specific cell cycle stages. We synchronized cells at the mitotic phase using the thymidine and nocodazole block method and released them from the arrest to enrich for cells at specific cell cycle phases for protein fractionation (Fig. 2A). As expected, we detected PCNA in the chromatin fraction only in S phase. However, RFC and RLC proteins did not change the fractionation profile by cell cycle stages. In particular, RFC1 and ATAD5 were detected only in the pellet fraction throughout the cell cycle. In cells arrested at prophase/prometaphase by nocodazole treatment, lamina disassembly and nuclear envelope breakdown are inhibited^44^, which might explain the presence of RFC/RLCs in the pellet fraction.

Notably, all large subunits of the RFC/RLCs displayed slightly up-shifted bands in the mitotic phase (Fig. 2A). The band shift disappeared after we treated the protein sample with phosphatase (Fig. 2B), indicating that all large subunits of the RFC and RLCs undergo phosphorylation during mitosis. This result is consistent with previous proteomics studies which show that several residues on RFC1, ATAD5, and CTF18 are highly phosphorylated in cells arrested at the G2/M phases compared to asynchronous cells^45,46^. Among the phosphorylation sites, serine 281 of RFC1 and serine 369 of ATAD5 are present in the consensus phosphorylation motif for aurora A kinase (AURKA) and aurora B kinase (AURKB), which are master mitotic kinases^47^.

We decided to investigate the serine 369 (pS369) phosphorylation site of ATAD5 further. We generated anti-pS369 ATAD5 antibody and then performed an in vitro aurora kinase assay with the purified ATAD5 fragment (residues 1–400) (Fig. 2C,D). We detected pS369-ATAD5 in reactions with wild-type (WT) ATAD5 and purified AURKA but not in reactions with kinase-dead AURKA or with the ATAD5 S369 to alanine (SA) substitution mutant (Fig. 2C). pS369-ATAD5 was also detected in reactions with purified AURKB but not in reactions with the ATAD5 SA mutant (Fig. 2D). In addition, pS369-ATAD5 disappeared when inhibitors for AURKA and AURKB, alisertib and barasertib, respectively, were included in the kinase reactions (Fig. 2C,D). These results suggest that ATAD5 is phosphorylated by either AURKA or AURKB.

Next, we examined the phosphorylation of ATAD5 at S369 in cells during mitosis. Phosphorylation of endogenous ATAD5 at S369 was observed in cells arrested at the G2/M phase by nocodazole treatment (Fig. 2E). When cells were released from G2 arrest by treatment of the CDK1 inhibitor RO-3306, they entered mitosis within 30 min as determined by mitosis marker pS10-histone H3 (Fig. 2F). Accordingly, pS369-ATAD5 intensity reached its maximum 30 min after release, gradually decreased, and then disappeared simultaneously with the pS10-histone H3 signal, suggesting that ATAD5 is phosphorylated at S369 in cells during mitosis. Mitotic phosphorylation of FLAG-tagged and endogenous ATAD5 at S369 was reduced by small interfering RNA (siRNA)-mediated depletion of AURKA or AURKB (Fig. 2G,H). Treatment with alisertib or barasertib alone slightly reduced pS369-ATAD5, and co-treatment with both drugs significantly reduced pS369-ATAD5 (Fig. 2I). These data suggest that ATAD5 at S369 is phosphorylated by two aurora kinases during mitosis. Lastly, we found that both the phosphorylation-defective ATAD5 S369A and the phosphomimetic S369E (S369 to glutamate) mutant proteins displayed a fractionation profile similar to that of wild-type ATAD5 (Supplementary Fig. 2), suggesting that the phosphorylation of ATAD5 at S369 is not required for association of ATAD5 with nuclease-resistant structures during mitosis.

### The fractionation profile of RFC/RLC proteins is not changed by DNA damage

Besides DNA replication during S phase, DNA synthesis also occurs during the DNA repair process, including base or nucleotide excision repair and homologous recombination of double-strand breaks (DSBs), which accompany PCNA loading and unloading by RFC and ATAD5-RLC, respectively^17^. RFC and ATAD5 rapidly move to ultraviolet laser-generated DNA lesions^48,49^. In budding yeast, Elg1 and Ctf18 move to DSB sites^50,51^. RAD17-RLC loads the 9-1-1 complex at DNA damage sites for checkpoint activation^23,24^.

**Figure 3:**
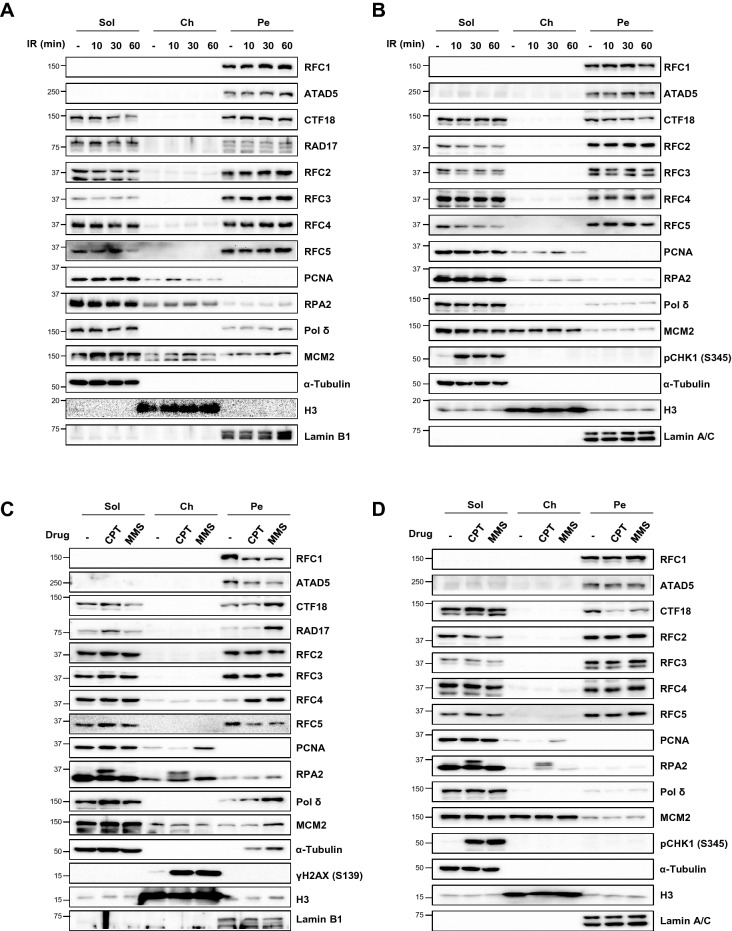
The fractionation profile of RFC/RLC proteins is not affected by DNA damage. (**A**, **B**) HeLa (**A**) or HEK293T (**B**) cells were irradiated with 10 Gy (Gy) X-ray and recovered for indicated times. (**C**, **D**) HeLa (**C**) or HEK293T (**D**) cells were treated 2 μM camptothecin (CPT) or 0.02% methyl methanesulfonate (MMS) for 2 h. (**A**–**D**) After treatment, cells were fractionated and each fraction was subjected to immunoblotting. (**A**–**D**) Uncropped blot images are presented in Supplementary Fig. 5.

Based on the involvement of RFC and three RLCs in the DNA damage repair pathway, we investigated whether the fractionation profile of RFC/RLC is affected by DNA damage using HeLa and HEK293T cells. We applied three different types of DNA damage: ionizing radiation (IR), camptothecin (CPT), and methyl methanesulfonate (MMS). The repair mechanisms for these types of damage, including homologous recombination, base excision repair (BER), and single-strand break repair (SSBR), each utilize DNA synthesis. None of the three types of DNA damage led to detection of RFC/RLC proteins in the chromatin fraction, and the overall fractionation profile remained the same in both cells (Fig. 3A–D). Treatment with CPT or MMS slightly reduced the amount of RFC1 and ATAD5 protein in the pellet fraction in HeLa but not in HEK293T cells (Fig. 3C,D). The most prominent change was the increase of RAD17 in the pellet fraction following MMS treatment (Fig. 3C), which is likely related to damage checkpoint activation. A recent report found that TopBP1 forms micrometer-sized condensates at DNA replication impediments and switches on ATR checkpoint signaling^52^. Considering the cooperative role of the RAD17-RLC-loaded 9-1-1 clamp with TopBP1 in checkpoint activation^23,24^, it is possible that the increased RAD17 in the pellet fraction co-exists with TopBP1 condensates.

### RFC4 and RFC5 are important for the protein stability of RFC/RLCs

**Figure 4:**
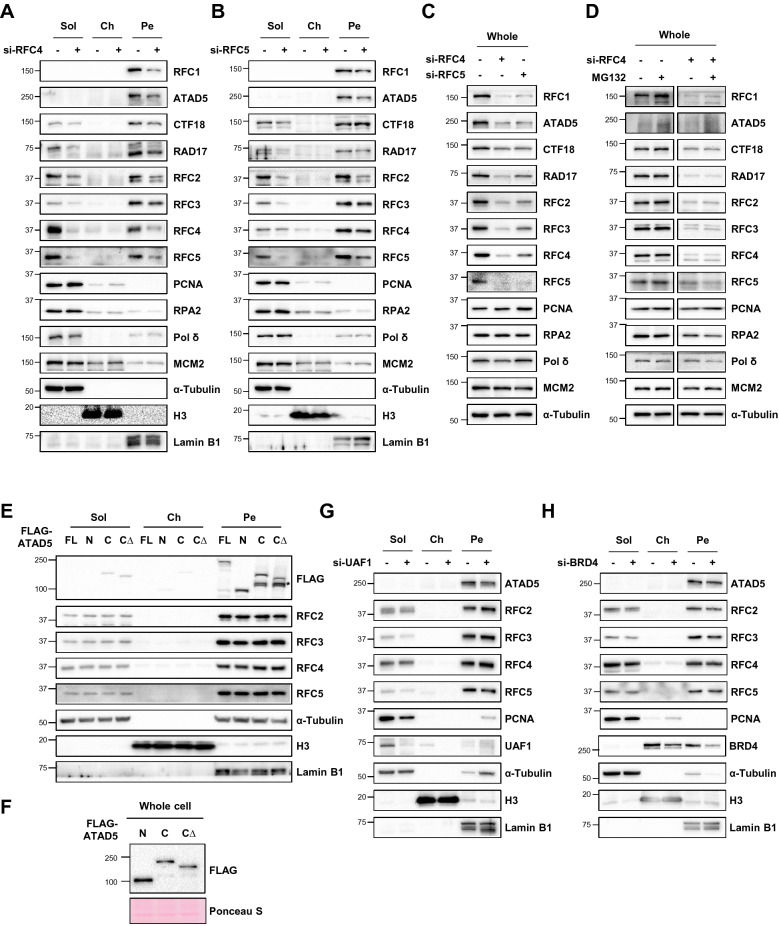
RFC4 and RFC5 maintain the protein levels of RFC/RLC proteins. (**A**–**D**) HeLa cells were transfected with siRNA as indicated. Forty-eight hours after transfection, cells were fractionated (**A**, **B**) or whole-cell extracts was prepared (**C**, **D**) for immunoblotting. (**D**) 10 μM MG132 was treated for 5 h before cell harvest. (**E**, **F**) HeLa cells were transfected with 3xFLAG-tagged full length ATAD5 (FL), N-terminal fragment (residues 1–693, N), C-terminal fragment (residues 694–1844, C) or small RFC interaction-defective C-terminal fragment (residues 694–1719, C∆) cDNA. Forty-eight hours after transfection, cells were fractionated (**E**) or whole-cell extracts were prepared (**F**), and then proteins were subjected to immunoblotting. (**E**) *, nonspecific band. (**G**, **H**) HeLa cells were transfected with siRNA as indicated and fractionated for immunoblotting. (**A**–**H**) Uncropped blot images are presented in Supplementary Fig. 5.

The RFC and three RLCs share small RFC subunits. Because all large subunits of the RFC/RLCs were detected in the pellet fraction and RFC4 bridges a large subunit to other small subunits in the RFC/RLCs^16^, we checked the effects of RFC4 depletion on the fractionation profile in HeLa and HEK293T cells. We found that RFC4 depletion reduced the level of all other RFC/RLC proteins, both in the soluble and pellet fractions (Fig. 4A and Supplementary Fig. 3A). Depletion of RFC5 also displayed a similar but less pronounced effect on other RFC/RLC proteins (Fig. 4B). This suggests that the protein levels of other subunits of RFC/RLC proteins are affected by the depletion of any small RFC subunit. When we checked the protein levels in whole cell extracts, depletion of RFC4 or RFC5 consistently reduced the protein amount of other RFC/RLC subunit proteins in both cell lines (Fig. 4C and Supplementary Fig. 3B). To check whether the reduction results from proteasomal degradation, we used a proteasome inhibitor and monitored protein levels of RFC/RLC subunits. Slight increase of total protein levels of RFC1 and ATAD5 by treatment of MG132 suggest that two proteins are regulated by proteasomal degradation pathway in unperturbed cells (Fig. 4D). However, the treatment did not restore total protein levels of RFC/RLC proteins reduced by RFC4 depletion (Fig. 4D). These results suggest that the reduction of RFC/RLC subunit proteins upon RFC4 depletion is due to destabilization of the RFC/RLCs rather than through active degradation mechanisms.

Because the small RFC subunits affected the protein amount of the large subunits, we investigated whether interaction between small and large subunits—in particular, the ATAD5—affects the fractionation profile of the large subunit. The large subunit ATAD5 directly interacts with a subset of proteins, including ubiquitin specific protease 1-associated factor (UAF1), chromatin reader bromodomain-containing protein 4 (BRD4), and recombinase RAD51, via the N-terminal region (residues 368–693)^39,53–55^. The C-terminal region of ATAD5 contains the ATPase domain (residues 1052–1412) and the small RFC interacting domain (residues 1602–1844). The ATAD5 C-terminal fragments (residues 694–1844) can unload PCNA both in vitro and in cells^19^. We examined the fractionation profile of the ATAD5 N-terminal fragment (residues 1–693, N), C-terminal fragment (residues 694–1844, C), and C-terminal fragment with a defect in the portion that interacts with the small RFC subunit (residues 694–1719, C∆). Even with slightly higher protein expression than the C-terminal fragment (Fig. 4F), the N-terminal fragment was still predominantly detected in the pellet fraction (Fig. 4E). Although both C-terminal fragments (C and C∆) were detected in the soluble and pellet fractions, protein levels of the C∆ fragment were much lower in the pellet fraction compared to the C-terminal fragment (Fig. 4E). These results suggest that, in the case of ATAD5, both N-terminal and C-terminal regions have the capacity to associate with the NM or chromatin inaccessible to nucleases, and the interaction with small RFC subunits contributes to, but is not required for, this association. Since the N-terminal fragment of ATAD5 was found in the pellet fraction, we examined the contribution of two ATAD5 interacting proteins, UAF1 and BRD4, to the retention of ATAD5 in the nuclease-resistant structures. Depletion of UAF1 or BRD4 did not change the fractionation profile of ATAD5 (Fig. 4G,H). These results suggest that there are other mechanisms to maintain association of the ATAD5 N-terminal fragment with nuclease-resistant structures.

### LAP2 interacts with ATAD5, RFC1 and RFC4

**Figure 5:**
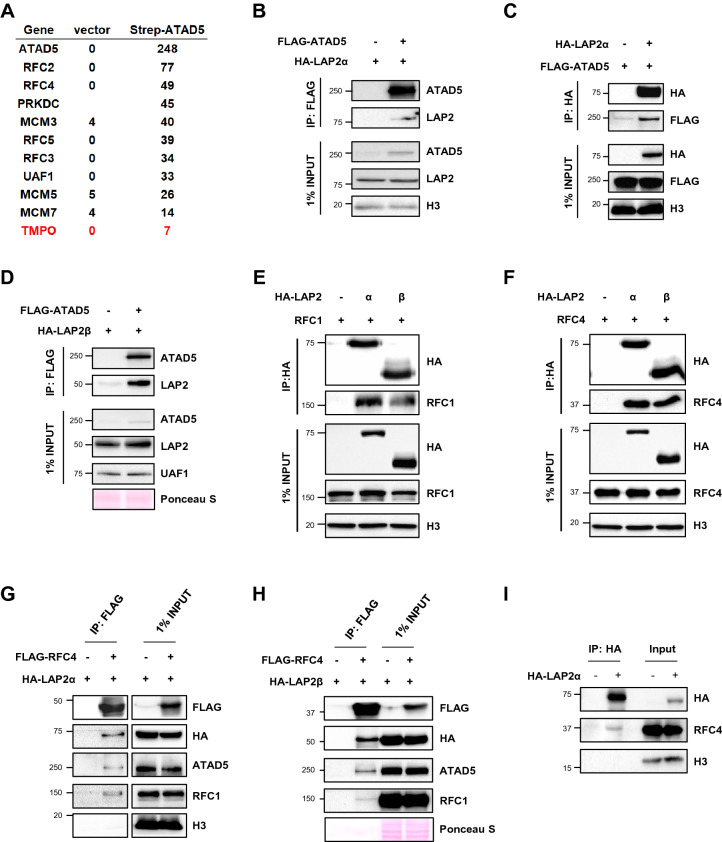
LAP2 interacts with the ATAD5, RFC1 and RFC4. (**A**) HEK293T cells were transfected with control Strep-tag II vector (vector) or Strep-tag II-tagged ATAD5 (Strep-ATAD5) cDNA. Forty-eight hours after transfection, proteins were extracted and subjected to affinity purification-mass spectrometry analysis. The numbers of peptide hits for some selected proteins from the analysis were displayed. Protein in red indicate TMPO gene products. (**B**, **C**, **D**) HEK293T cells were co-transfected with 3xFLAG-tagged ATAD5 and HA-tagged LAP2α (**B**, **C**) or LAP2β (**D**) cDNA. Whole-cell extracts were immunoprecipitated with anti-FLAG (**B**, **D**) or anti-HA (**C**) antibody followed by immunoblotting. (**E**, **F**) HEK293T cells were co-transfected with HA-tagged LAP2α or LAP2β and RFC1 or RFC4 cDNA. Whole-cell extracts were immunoprecipitated with anti-HA antibody followed by immunoblotting. (**G**, **H**) HEK293T cells were co-transfected with HA-tagged LAP2α or LAP2β and 3xFLAG-tagged RFC4 cDNA. Whole-cell extracts were immunoprecipitated with anti-FLAG antibody followed by immunoblotting. (**I**) HEK293T cells were transfected with HA-tagged LAP2α cDNA. Whole-cell extracts were immunoprecipitated with anti-HA antibody followed by immunoblotting. (**B**–**I**) Uncropped blot images are presented in Supplementary Fig. 5.

To find the molecular requirements for the association of RFC/RLCs with nuclease-resistant structures, we identified proteins that interact with the RFC/RLC proteins. We conducted affinity purification-mass spectrometry analysis using the immunoprecipitates of Strep-tag II tagged-ATAD5 expressed in cells^56^. The analysis revealed the thymopoietin (TMPO) gene product as the putative ATAD5 interacting protein (Fig. 5A). Two major TMPO gene products are LAP2α and β^57–59^. LAP2α is present in the nucleoplasm and is associated with intra-nuclear lamin A/C. LAP2β is a transmembrane protein that is incorporated into the nuclear inner membrane and the nuclear laminar structure and is thought to be important for chromatin association with the lamina.

We first confirmed the protein–protein interactions between ATAD5 and LAP2α and β. Immunoprecipitation of FLAG-ATAD5 pulled down HA-LAP2α and HA-LAP2β (Fig. 5B,C). Immunoprecipitation of HA-LAP2α also pulled down FLAG-ATAD5 (Fig. 5D). In addition, immunoprecipitation of either HA-LAP2α or HA-LAP2β pulled down RFC1 in cells transiently overexpressing RFC1 (Fig. 5E). Because RFC and ATAD5-RLC share the small RFC subunits, we also checked protein–protein interactions between RFC4 and LAP2. Immunoprecipitation of either HA-LAP2α or HA-LAP2β pulled down RFC4 in cells transiently overexpressing RFC4, and immunoprecipitation of FLAG-RFC4 pulled down HA-LAP2α and HA-LAP2β (Fig. 5F–H). Based on the association of LAP2 proteins with the NM, these results suggest that LAP2α and β may contribute to the association of RFC and ATAD5-RLC with the NM. We tried to detect interactions between endogenous LAP2α and ATAD5, RFC1 or RFC4, but with no success. In an alternative way, we found that overexpressed HA-tagged LAP2α pulled down endogenous RFC4 (Fig. 5I). Even in this trial, we could not detect ATAD5 or RFC1. We speculate that the interactions are not strong or very small fractions of protein interact with each other.

### LAP2α deficiency reduces the association of RFC/RLC proteins with nuclease-resistant structures

**Figure 6:**
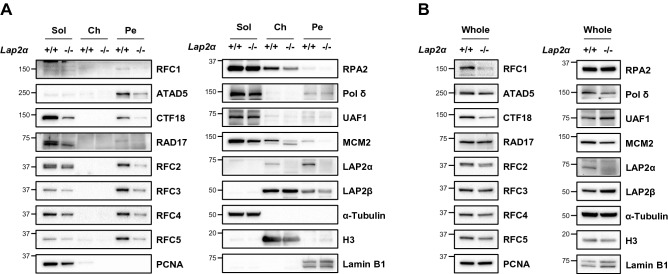
The association of RFC/RLC proteins with nuclease-resistant structures is reduced in *Lap2α* knockout cells. (**A**) *Lap2α*^+/+^ and *Lap2α*^−/−^ mouse skin fibroblasts were fractionated and each fraction was subjected to immunoblotting. (**B**) Whole-cell extracts from *Lap2α*^+/+^ and *Lap2α*^−/−^ mouse skin fibroblasts were subjected to immunoblotting. (**A**, **B**) Uncropped blot images are presented in Supplementary Fig. 5.

We examined the effects of LAP2α depletion on the fractionation profile of RFC/RLCs using *Lap2α* knockout mouse dermal fibroblast cells^60^. We found that the protein levels of all RFC/RLC proteins in the pellet fraction were reduced in the *Lap2α* knockout cells (Fig. 6A). LAP2α deficiency reduced the protein levels of most RFC/RLC proteins in the soluble fraction, similar to RFC4 depletion, but the reduced amount was lower compared to RFC4 depletion. In the RFC4-depleted cells (Fig. 4A), the degree of reduction of RFC/RLC proteins was larger in the soluble fraction than in the pellet fraction. However, in *Lap2α* knockout cells (Fig. 6A), the degree of reduction of RFC/RLC proteins was milder in the soluble fraction than the pellet fraction. Consistently, the effects of LAP2α depletion on total protein level of the RFC/RLC proteins was low and variable compared to the pleiotropic effects of RFC4 depletion (Fig. 6B). Only RFC1 and CTF18 showed a marked reduction in the *Lap2α* knockout cells, while other RFC/RLC proteins decreased only marginally (Fig. 6B). These results suggest that the association of the RFC/RLC proteins with the nuclease-resistant structures is reduced in the *Lap2α* knockout cells.

We also examined the effects of LAP2α depletion on the fractionation profile of RFC/RLCs in HeLa cells. We could not see any differences in the fractionation profiles of the RFC/RLC proteins by one-time LAP2ɑ siRNA transfection. When LAP2ɑ was depleted for a longer time, a clear reduction in the pellet fraction and total protein level of RFC1 was observed (Supplementary Fig. 4A,B). While slight reduction of ATAD5 and CTF18 in the pellet fraction was also observed in the LAP2ɑ-depleted cells, the fractionation profiles of other RFC/RLC proteins were not significantly changed. It cannot be excluded that the reduction in RFC1 in LAP2ɑ-depleted HeLa cells results from alteration in nuclear matrix as observed by reduction of Lamin B1 in the pellet fraction and whole cell extracts although the fractionation profiles of another nuclear matrix protein, Emerin was not changed. Overall, the effect of LAP2ɑ depletion by siRNA in HeLa cells was marginal compared to Lap2α knockout mouse fibroblast cells.

## Discussion

In this report, we performed subcellular fractionation to identify structural localization of RFC/RLC and other replication-related proteins. Unexpectedly, we detected RFC1 and ATAD5 only in the nuclease- and high salt-resistant fraction, while small RFC proteins were located in both soluble and pellet fractions (Fig. 1C and Supplementary Fig. 1), which were independent of cell cycle and DNA damage (Figs. 2, 3). PCNA performs its role as a processivity factor and as a binding platform while encircling chromatin DNA^14^. PCNA loading and unloading is closely associated with chromatin during DNA replication and repair. It has been assumed that the RFC and ATAD5-RLC might be recruited in a timely manner from the nucleoplasmic pool to replication forks or damaged sites or that they are at least associated with chromatin at the time of their action. However, neither RFC1 nor ATAD5 were detected in the soluble or chromatin fractions in our fractionation procedure, which employed the widely used CSK extraction and nuclease treatment (Fig. 1C and Supplementary Fig. 1). We tested the possibility that these results were artifactual by modifying our extraction procedure and checking the fractionation profile of exogenously expressed RFC1 and ATAD5. After carefully minimizing the possibility of experimental artifacts using different fractionation methods, we strongly believe that most RFC and ATAD5-RLC proteins are primarily associated with nuclease-resistant chromatin and/or NM.

The ATP-bound RFC complex binds to and opens a PCNA homotrimer ring, and the loading of the RFC-ring-opened PCNA intermediates onto DNA triggers the release of PCNA from the RFC complex with closure of the PCNA ring to encircle the DNA^14,15^. PCNA unloading occurs in the opposite direction. The ATP-bound ATAD5-RLC opens the PCNA ring, and the ATAD5-RLC-PCNA intermediates are then released from the DNA^19^. Our results suggest that these processes occur where the related proteins are highly accumulated in the chromatin and/or while being tightly associated with the stationary structure because nuclease treatment and high salt treatment cannot extract them. Replication foci observed during the S phase could be highly enriched for RFC/RLC proteins. In a replication factory, many proteins, including RFC and ATAD5-RLC, participate in chromatin replication^13^. Detection of MCM2, FEN1, and pol δ in the nuclease-resistant fractions also support the protein-dense cooperative replication process at specific locations in the nucleus (Fig. 1J and Supplementary Fig. 1). Although it is not yet known whether the replication machinery is anchored to stationary structures such as the nuclear matrix, a budding yeast study showing the passage of replicating DNA through the stationary replication factory supports our model^36^. The spatial integration of replication proteins might be beneficial for simultaneous firing of adjacent origins and efficient replication in each replication domain.

Based on our results, the RFC and ATAD5-RLC are associated with nuclease-resistant structures regardless of cell cycle phase. PCNA loading and unloading occur during DNA repair synthesis in all cell cycle phases. All subunits of the human RFC complex and the budding yeast Elg1 rapidly increase their signals at DNA lesions, including DSBs, independent of cell cycle^48,49,51^. It is possible that the RFC-ring-opened PCNA intermediates are anchored to these nuclease-resistant structures in a ‘ready-for-PCNA loading’ mode in order to rapidly load PCNA at any double-strand DNA/single-strand DNA junction with a 3`-hydroxyl group. This mode of PCNA loading might also occur during DNA replication, especially in lagging strand synthesis, which accompanies frequent PCNA cycling. Further studies are needed to determine whether the association of pre-formed RFC-ring-opened PCNA intermediates with the stationary structure in itself is beneficial for PCNA loading. We speculate that the RFC-ring-opened PCNA intermediates are part of protein-dense aggregates maintained by protein–protein interactions between replication/repair proteins and their association with stationary structures, and this may facilitate the replication/repair process while synergizing with other proteins.

We identified LAP2 proteins as putative ATAD5 interacting proteins and confirmed interactions between ATAD5 and LAP2α and β (Fig. 5). RFC1 and RFC4 also interacted with LAP2α and β. Among several LAP2 isoforms, LAP2α is distinctive in that it is the only LAP2 isoform that is present in the nucleoplasm^59^. LAP2α is associated with the lamin A/C in the nuclear interior and regulates the mobility of lamin A/C^1,9^. Based on our new findings and other reported roles of LAP2α, we hypothesized that LAP2α could be a linker required for the association of the RFC/RLCs with the intra-nuclear lamin A/C. Analysis of *Lap2α* knockout mouse cells showed a clear reduction of the RFC/RLC proteins in the pellet fraction but mild reduction in the soluble fraction (Fig. 6A). In addition, the effect of *LAP2α* knockout on total protein levels of the RFC/RLC proteins was marginal except RFC1 and CTF18 (Fig. 6B). Considering that the soluble fractions contained four times the amount of total protein compared to the pellet fraction, these results suggest that LAP2α contributes to the association of the RFC/RLC proteins with nuclease-resistant structures rather than contributing to the maintenance of the total protein levels of RFC/RLC, similar to the role of small RFC proteins. Compared to *Lap2α* knockout mouse fibroblast cells, the effect of siRNA-mediated LAP2ɑ depletion on the fractionation profile of the RFC/RLC proteins was marginal in HeLa cells (Supplementary Fig. 4A,B). Considering that the minimal changes were only observed after 6 days of transfection, it may require time for the effects of LAP2ɑ depletion to appear in HeLa cells, as evident in *Lap2α* knockout mouse cells. Another possibility is that the different degree of effect may be due to intrinsic differences between normal fibroblasts and cancer cells. For example, LAP2α is overexpressed in various human tumors and cancer cell lines^61^, which may attenuate the effects of transient LAP2ɑ depletion in HeLa cells.

Based on our study, the RFC/RLC proteins are likely associated with nuclease-resistant chromatin and/or nuclear matrix. Extraction of the RFC/RLC proteins from the pellet fraction by adding a sonication step supports the chromatin association hypothesis to some extent (Fig. 1G). Protein–protein interactions between the RFC/RLC proteins and LAP2 proteins and reduced protein levels of the RFC/RLC in pellet fractions due to LAP2α deficiency are consistent with NM association; however, because protein–protein interactions cannot be guaranteed at such high salt (2 M NaCl) wash conditions, there may be other factors at play. Our study did not show the degree of contribution of the two structures (chromatin and nuclear matrix) to the presence of RFC/RLC proteins in the pellet fraction. In addition, it is not clear whether the association of RFC/RLCs with chromatin and nuclear matrix occurs in the same physical place or not. Considering there is still no convincing evidence that a filamentous nuclear skeleton (representing the nuclear matrix) exists in living cells^62^, more technically advanced approaches will be required to elucidate these missing parts.

It has been reported that cell proliferation was increased in *Lap2α* knockout cells^60,63^. Considering the reduced protein level and association with nuclease-resistant structure of the RFC/RLC proteins by LAP2α deficiency (Fig. 6), the reported hyper-proliferation was different from what was expected. We speculate that multiple cellular functions of LAP2α generates complex outcomes. LAP2α regulates the G1 to S cell cycle transition by suppressing the RB/E2F pathway^63^, which might explain dysregulated proliferation by LAP2α deficiency. However, how LAP2α regulates RFC/RLC proteins and the biological implications of this regulation still remains to be investigated.

RFC1 is essential for cell survival in eukaryotes, while the other large subunits of RLCs are not essential for cell viability in yeast and mammals (although some human cancer cells depend on each gene for survival)^64–67^. All small RFC subunits are essential for cell survival^65,66^, and they contribute to the activity of the RFC/RLCs in different ways. When the RFC/RLCs bind to the PCNA clamp or the 9-1-1 checkpoint clamp, the small RFC proteins provide physical contacts with the clamp^68,69^. The ordered hydrolysis of ATP in the RFC subunits, including the small RFC proteins, is required for PCNA loading^16^. Here, we found that the small RFC subunits can also contribute to the maintenance of overall protein levels of RFC/RLCs (Fig. 4C and Supplementary Fig. 3). The protein stability of the multi-subunit complex comprised of AAA + ATPase subunits is severely affected by deficiency of a subunit^70^. Since RFC and RLCs are also AAA + ATPase complexes, the destabilization of the large subunits upon depletion of the small RFC subunits occurs in a similar way rather than using active degradation mechanisms, such as proteasomal degradation (Fig. 4D). It has been recently reported that many protein complexes are cotranslationally assembled in eukaryotes and it can prevent degradation of subunit prone to aggregation^71^. Since the RFC interaction domain is located at C-terminal in ATAD5 and all five RFC subunit^19,68^, rather than cotranslational subunit interaction, complex assembly right after ribosome dissociation might be more probable for the assembly of the RFC/RLC complex.

All large subunits of RFC and RLCs undergo phosphorylation during mitosis (Fig. 2A,B). This is consistent with previously published proteomics studies that showed phosphorylation of RFC1, ATAD5, and CTF18 in cells during G2/M phases^45,46^. In this report, we showed that ATAD5 was phosphorylated at S369 by two aurora kinases in vitro and in the cells during mitosis (Fig. 2). Future studies should identify responsible kinases, regulation, and biological significance of the mitotic phosphorylation of the large subunits of the RFC and RLCs. The phosphorylation of ATAD5 at S369 might be related with regulation of specific cellular processes by ATAD5 but not the association of ATAD5 with nuclease-resistant structures (Supplementary Fig. 2). ATAD5 localizes to the centrosome, the primary microtubule organizing center, where it regulates centrosome duplication^72^. AURKA also localizes to centrosomes and is involved in centrosome separation, duplication, and maturation as well as in bipolar spindle assembly^73^. Therefore, the relationship between ATAD5 phosphorylation at S369 and centrosome regulation should be investigated further. The S653 residue of ATAD5 is also reportedly phosphorylated by the mitotic cyclin-dependent kinase, which inhibits the interaction between ATAD5 and BRD4^53,55^. Our preliminary mass spectrometry analysis also identified another pleiotropic master mitotic kinase, Polo-like kinase 1, as an ATAD5-interacting protein (data not shown). These data suggest that ATAD5 is phosphorylated at multiple residues by different kinases during mitosis, which might regulate specific biological processes.

The most unexpected finding in our study was that neither RFC1 nor ATAD5 were detected in the soluble or nuclease-susceptible chromatin fractions. A previous study found that RFC1 was not present in the soluble fraction but was instead bound to chromatin after fractionation based on the method of Mendez and Stillman^48,74^. However, this paper did not test whether RFC1 was eluted from the pellet (expected to contain proteins associated with chromatin or nuclear matrix) upon nuclease treatment^48^. Irrespective of whether the RFC/RLCs are associated with the chromatin or NM, the rapid accumulation of RFC/RLC proteins at DNA damaged sites requires a mechanical explanation that encompasses the subcellular localization of the RFC/RLCs, even with the detection of RFC1 only in the nuclease-resistant fractions in our study. With the advent of new technologies, it will be worth investigating whether the ‘ready-for-PCNA loading’ mode by the RFC complex associated with the stationary structures is actually utilized during DNA replication and repair.

## Methods

### Cell lines, cell culture and cell synchronization

Human embryonic kidney (HEK) 293 T and HeLa cells were purchased from ATCC and cultured in Dulbecco’s modified Eagle’s medium (DMEM) supplemented with 10% fetal bovine serum (GE Healthcare), 100 U/mL penicillin G (Life Technologies), and 100 μg/mL streptomycin (Life Technologies) at 5% CO_2_, 37 °C. Immortalized skin fibroblasts from *Lap2α* knockout mice and wild-type littermates were kindly provided by Dr. Foisner and were cultured as described^60^. To enrich cells at the M phase, cells were treated with 100 ng/ml nocodazole for 12 h and collected. To obtain cells at G1 and S phases, cells were arrested at the M phase by the thymidine-nocodazole block (2 mM thymidine for 24 h, washing, incubation in fresh media for 3 h, and 100 ng/ml nocodazole for 12 h), shaken off the plate, collected, washed with culture medium and incubated in fresh media. To synchronize cells at the G2 phase, cells were treated with 10 μM CDK1 inhibitor RO-3306 for 16 h.

### Chemicals and antibodies

The following drugs were used in this study: Alisertib, Barasertib (Selleckchem), RO-3306, nocodazole, thymidine, methyl methanesulfonate, camptothecin, MG-132 (Sigma-Aldrich). The following antibodies were used in this study: anti-RAD17, anti-PCNA (PC10), anti-RFC4, anti-RFC5, anti-UAF1, anti-LAMIN B1, anti-Emerin, and anti-Lamin A/C antibodies (Santa Cruz Biotechnology), anti-MCM2, anti-RFC2, anti-RFC3, anti-Aurora A kinase, anti-Aurora B kinase, anti-LAP2, and anti-LAP2α antibodies (Abcam), anti-RFC1 antibody (Novus Biologicals), anti-CDT1, anti-phospho histone H3 (ser10) antibodies (Cell signaling Tech), anti-phospho MCM2 (S40/41), anti-CTF18, anti-RPA2 antibodies (Bethyl Laboratories), anti-DNA polymerase δ antibody (BD Biosciences), anti-histone H3 and anti-phospho H2AX antibodies (Thermo Fisher Scientifics), anti-FLAG, anti-HA, anti-α-tubulin antibodies (Sigma-Aldrich). The anti-human ATAD5 antibody was raised in rabbits using the N-terminal fragment (Residues 1–297 amino acids)^54^. The anti-human anti-pS369 ATAD5 antibody was generated in rabbits by using the pS369 synthetic ATAD5 peptide (AbClon Inc.).

### Plasmids, small interfering RNAs (siRNAs), and transfection

LAP2α cDNA was retrieved from the Human HeLa QUICK-Clone cDNA (Clontech) by polymerase chain reaction. LAP2β cDNA was purchased from Addgene. Both cDNA was cloned into the pcDNA5/FRT/TO vector (Thermo Fisher Scientifics) together with HA-tag sequence in the N-terminus region. The siRNA sequences used in this study were: ATAD5 3′UTR (5′-GUAUAUUUCUCGAUGUACA-3′); UAF1 (5′-AAUCAGCACAAGCAAGAUCCAUAUA-3′); RFC4 (5′-AAGAGAUUAGGAAGAUCUG-3′); LMNA (5′-CUUACCGGUUCCCACCAAA-3′); LAP2α (5′-GCACAGAUUCUUAGCUCAGAU-3′); SMART pool siRNAs for AURKA, AURKB, RFC5 were used (Dharmacon). Pan BRD4 siRNA (siRNA ID: HSS141059) was used (Invitrogen). Transfections of plasmid DNA and small interfering RNAs (siRNAs, 20 nM), were performed using X-tremeGENE HP (Roche) and RNAiMAX (Thermo Fisher Scientific) respectively, according to the manufacturer’s instructions. Transfection reagents was removed 6 h post transfection and fresh medium was added. Cells were analyzed at 48 h after transfection.

### Subcellular fractionation

Subcellular fractionation was performed as described in^37^ with slight modification. 2.5 × 10^6^ HeLa cells were incubated in 150 μl CSK buffer (10 mM PIPES, 100 mM NaCl, 300 mM sucrose, 2 mM MgCl_2_, 1 mM EGTA, and 0.5% Triton X-100) for 5 min at 4 °C followed by centrifugation. Supernatant was collected as ‘soluble fraction’ while pellet was washed in CSK buffer and was further lysed with 35 μl CSK buffer supplemented with Benzonase nuclease (250 U/mL) and 5 mM MgCl_2_ for 20 min at 37 °C. Then, a final concentration of 0.25 M ammonium sulfate was added for 5 min at 4 °C followed by centrifugation to obtain ‘chromatin fraction’. Remaining nuclease-resistant pellet was washed with high-salt-CSK buffer (CSK buffer with 2 M NaCl) for 5 min at 4 °C followed by centrifugation. Left-over nuclease- and high salt-resistant ‘pellet fraction’ was solubilized in 40 μl Reagent 3 (Bio-Rad laboratories) containing 5 M urea and 2 M thiourea. The ratio of total protein extracted for each fraction was about 4:1:1 (413.0 ± 47.2, 97.2 ± 11.9 and 94.2 ± 8.8 for soluble, chromatin, and pellet fractions; mean ± standard deviation, μg). For immunoblotting, we used each protein fraction at a ratio of 2:1:1.

### Whole-cell extracts preparation

Whole-cell extracts were prepared by cell pellet lysed with RIPA buffer (50 mM Tris–HCl pH 8.0, 150 mM NaCl, 5 mM EDTA, 1% Triton X-100, 0.1% sodium dodecyl sulfate, 0.5% sodium deoxycholate) supplemented with 0.1 M phenylmethylsulfonyl fluoride, PhosSTOP Phosphatase Inhibitor Cocktail (Roche), cOmplete Protease Inhibitor Cocktail (Roche) and Benzonase nuclease for 40 min on ice followed by sonication and centrifugation.

### Immunoprecipitation and immunoblot analysis

For immunoprecipitation, cells were lysed in ice-cold buffer X (100 mM Tris–HCl, 250 mM NaCl, 1 mM EDTA, 1% NP-40) supplemented with 0.1 M phenylmethylsulfonyl fluoride with PhosSTOP Phosphatase Inhibitor Cocktail (Roche), cOmplete Protease Inhibitor Cocktail (Roche), and Benzonase nuclease followed by sonication and centrifugation. Target proteins within the lysate were immunoprecipitated with indicated antibodies. For immunoblot analysis, proteins were separated by SDS-PAGE and transferred to a nitrocellulose membrane. Membranes were incubated in Tris-buffered saline containing 0.1% Tween 20 supplemented with 5% skim milk powder for blocking. Membranes were then cut according to protein sizes and incubated with primary antibodies for overnight. After washing, the blot was incubated with horseradish peroxidase-conjugated secondary antibodies (Enzo Life Sciences). Signals were detected using enhanced chemiluminescence reagent (Thermo Fisher Scientific) and an automated imaging system (ChemiDoc MP, Bio-Rad Laboratories). After image acquisition, antibody was stripped using the Restore western blot stripping buffer (Thermo Fisher Scientifics) and then reprobed with the next antibody.

### Protein purification

Human ATAD5 proteins for in vitro kinase assay were purified from *E. coli* cells. 1 L culture was grown to OD of 0.6, and 1 M isopropyl β-D-1-thiogalactopyranoside (IPTG) was added for 4 h to express protein. *E. coli* cells were centrifuged and resuspended in buffer A (20 mM Tris–HCl pH 8.0, 150 mM NaCl, 2 mM β–mercaptoethanol) with a cOmplete Protease Inhibitor Cocktail (Roche). Cells were lysed with lysozyme for 1 h at 4 °C and cell lysates were cleared by ultracentrifugation (13,000 rpm, 30 min). Proteins were purified by sequential application of cOmplete HIS-Tag resin (Roche), and ion exchange chromatography. The purified AURKA, AURKB and CDK1/cyclin B1 recombinant proteins were purchased from Millipore.

### The in vitro kinase assay

For the AURKA assay, 2 μg of purified ATAD5 protein was incubated with 100 ng of AURKA in the kinase buffer (500 mM Tris–HCl pH 7.4, 5 mM Na_3_VO_4_, 150 mM MgCl_2_, 10 mM DTT, 20 mM EGTA, 1 mM ATP) at 22 °C for 2 h. For the AURKB assay, 20.32 ng of AURKB together with 43.2 ng of CDK1/cyclin B1 was used at 30 °C for 2 h.

### Image acquisition and image analysis

Cells plated on LabTek™ chamber slides (Thermo Fisher Scientific) were treated with CSK buffer and/or Benzonase nuclease, washed with PBS, and fixed with 4% paraformaldehyde for 20 min at room temperature. After washing, cells were mounted using ProLong Gold antifade reagent (Vector Laboratories, Burlingame, CA, USA). Confocal images were acquired using an LSM880 confocal microscope (Carl Zeiss) with a 40 × /1.2 objective lens. Image acquisition and analysis were performed with Zen 2.6 (blue edition) (Carl Zeiss) software.

## Supplementary Information

Supplementary Information.
