# Supplementary material for: Characterization of subcellular localization of eukaryotic clamp loader/unloader and its regulatory mechanism
Source: Sci Rep. 2021 Nov 8;11:21817. doi: 10.1038/s41598-021-01336-w (PMC8575788; doi:10.1038/s41598-021-01336-w)
Supplement: Supplementary file 1 — Supplementary Information. [file 41598_2021_1336_MOESM1_ESM.pdf]

Supplementary Information

Characterization of subcellular localization of eukaryotic clamp loader/unloader and its regulatory mechanism

Park et al.

Supplementary Figure

Supplementary Figure 1

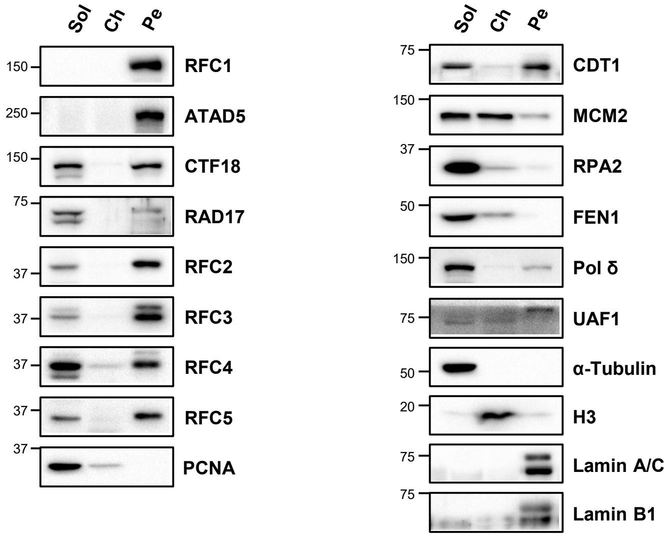

Supplementary Figure 1. (related to Figure 1) HEK293T cells were fractionated as the scheme in Figure 1A. Soluble (Sol), chromatin (Ch) and pellet (Pe) fractions were subjected to immunoblotting as indicated. Uncropped blot images are presented in Supplementary Figure 5 .

Supplementary Figure 2

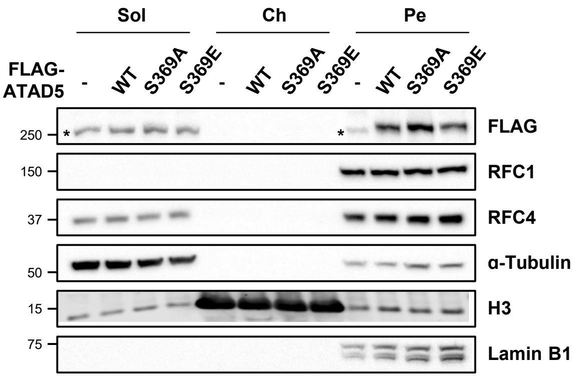

Supplementary Figure 2. (related to Figure 2) HeLa cells were transfected with cDNA expressing 3xFLAG-tagged wild-type (WT) ATAD5, ATAD5 S369A or S369E mutant. Forty-eight hours after transfection, cells were fractionated for immunoblotting. *, nonspecific bands. Uncropped blot images are presented in Supplementary Figure 5 .

Supplementary Figure 3

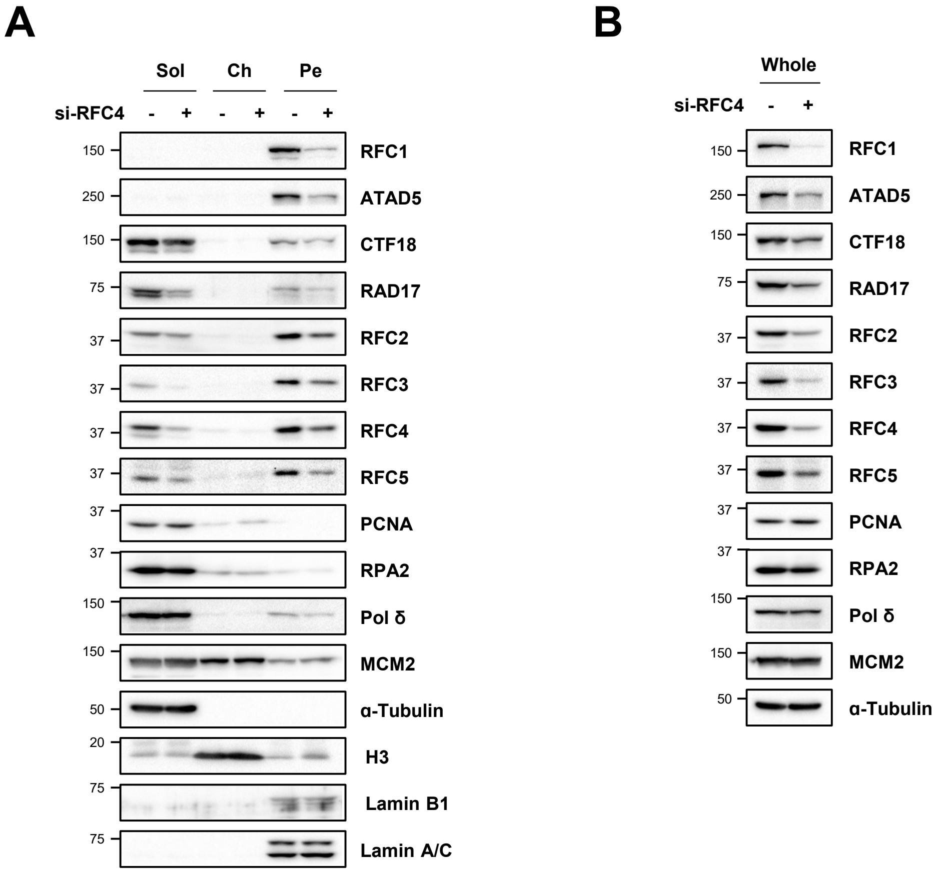

Supplementary Figure 3. (related to Figure 4A-C) (A, B) HEK293T cells were transfected with control or *RFC4* siRNA. Forty-eight hours after transfection, cells were fractionated (A) or whole-cell extracts was prepared (B) for immunoblotting. Uncropped blot images are presented in Supplementary Figure 5 .

Supplementary Figure 4

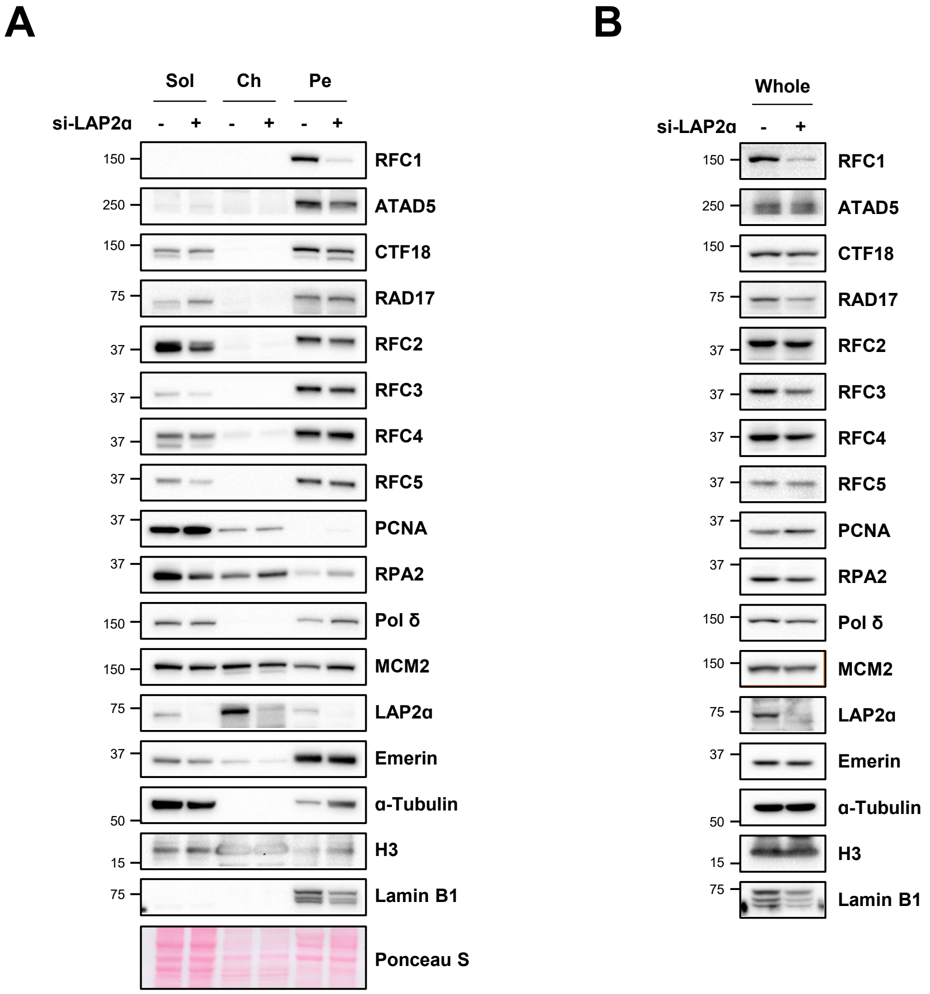

Supplementary Figure 4. (related to Figure 6A and B). HeLa cells were transfected with control or *LAP2α* siRNA twice at 3-day intervals. Two days after the second transfection, cells were fractionated (A) or whole-cell extracts was prepared (B) for immunoblotting. Uncropped blot images are presented in Supplementary Figure 5 .

Supplementary Figure 5

1B

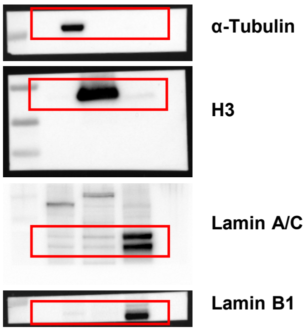

1C

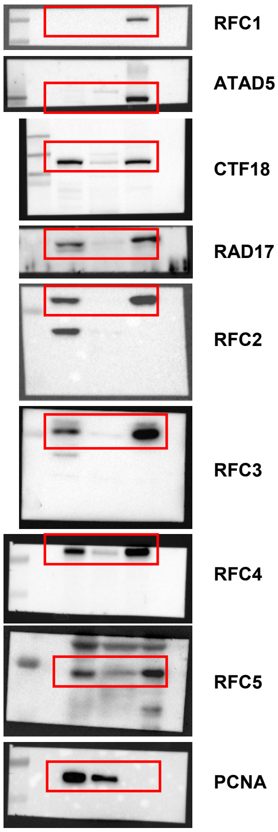

1D

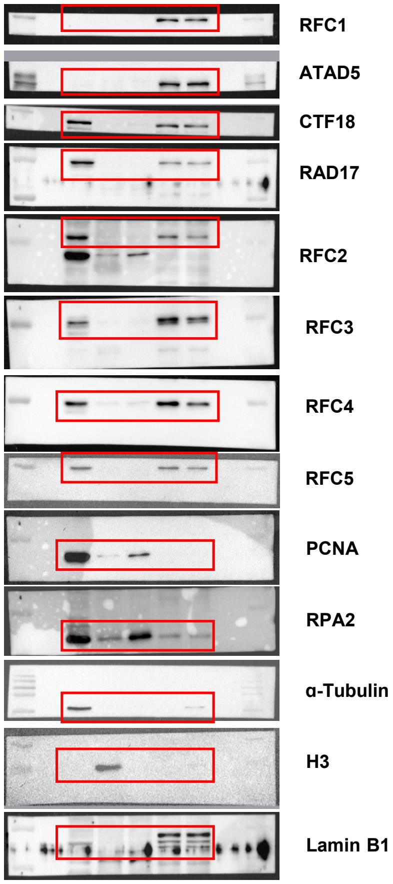

1E

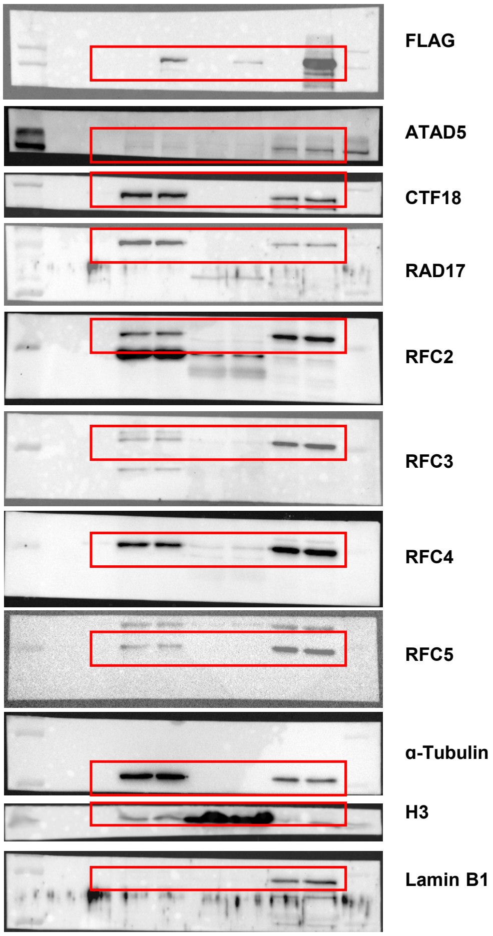

1F

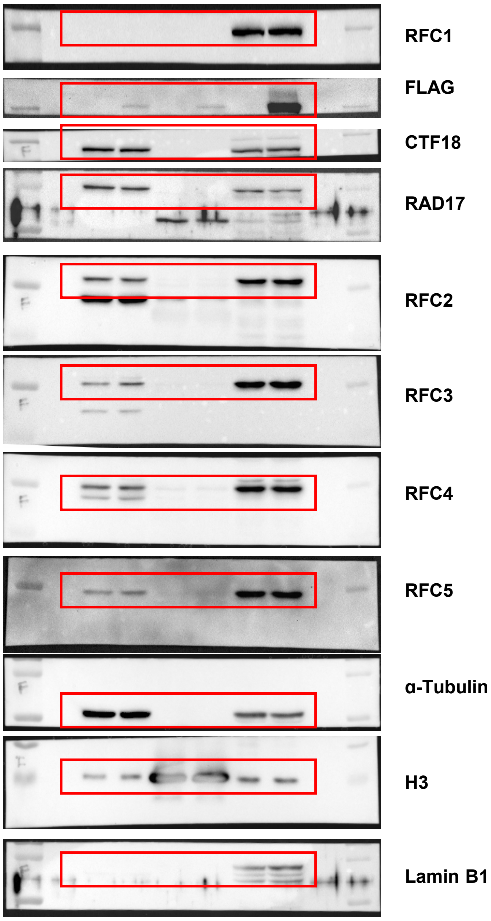

Supplementary Figure 5 (continued)

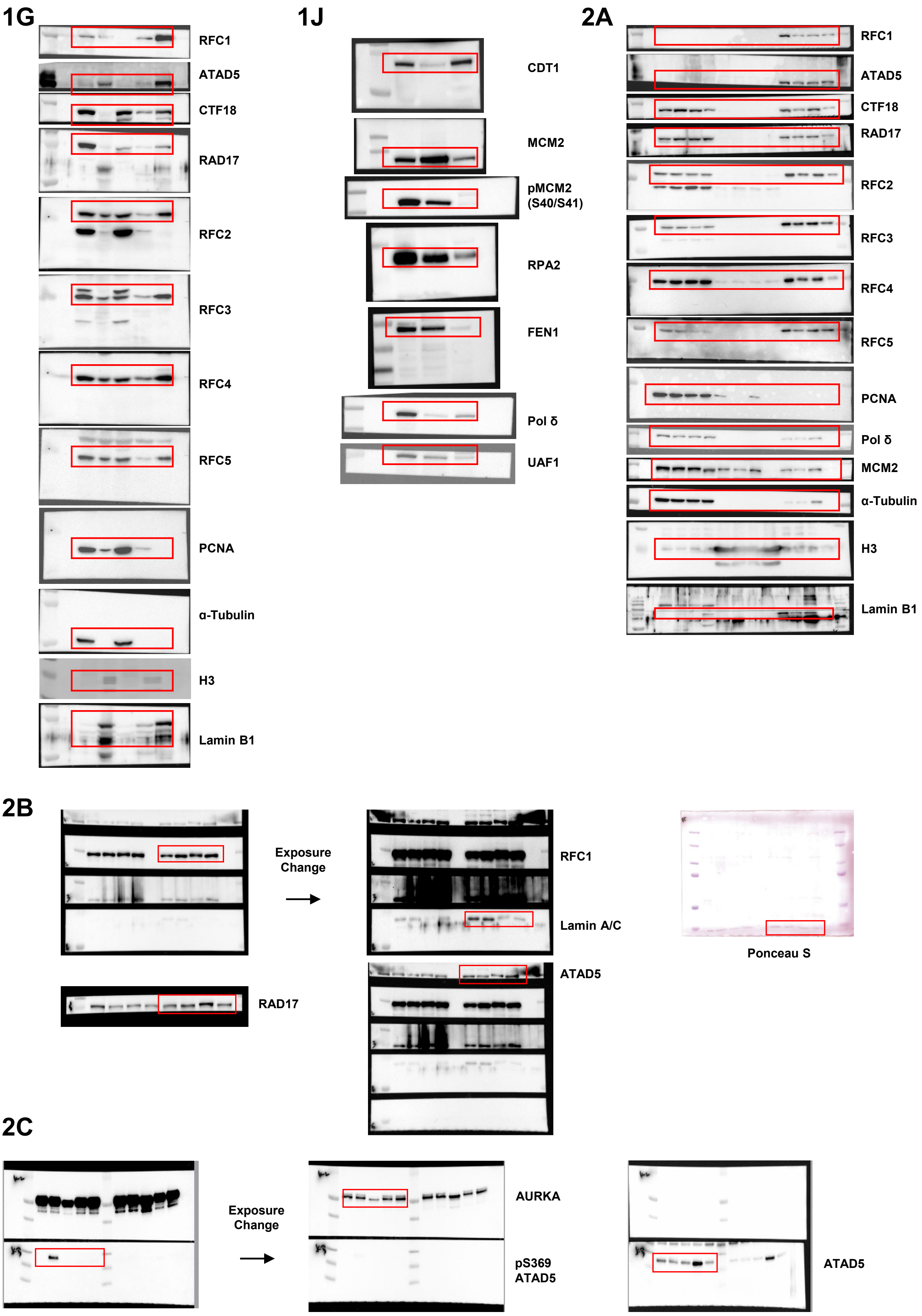

Supplementary Figure 5 (continued)

2D

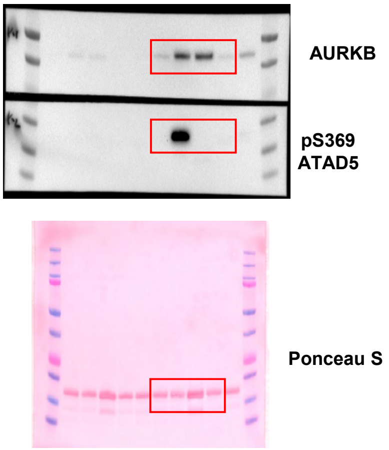

2E

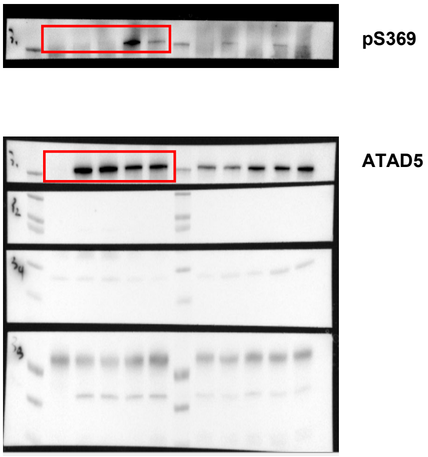

2F

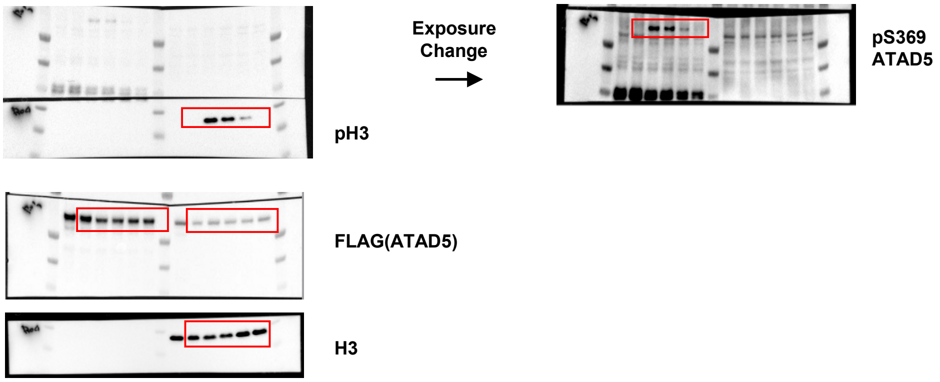

2G

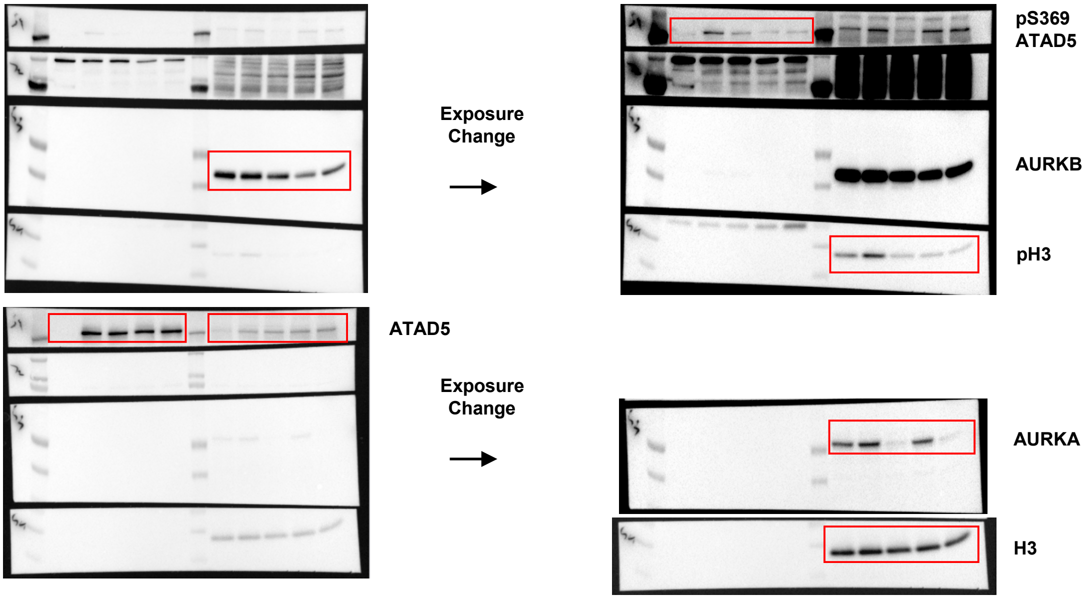

Supplementary Figure 5 (continued)

2H

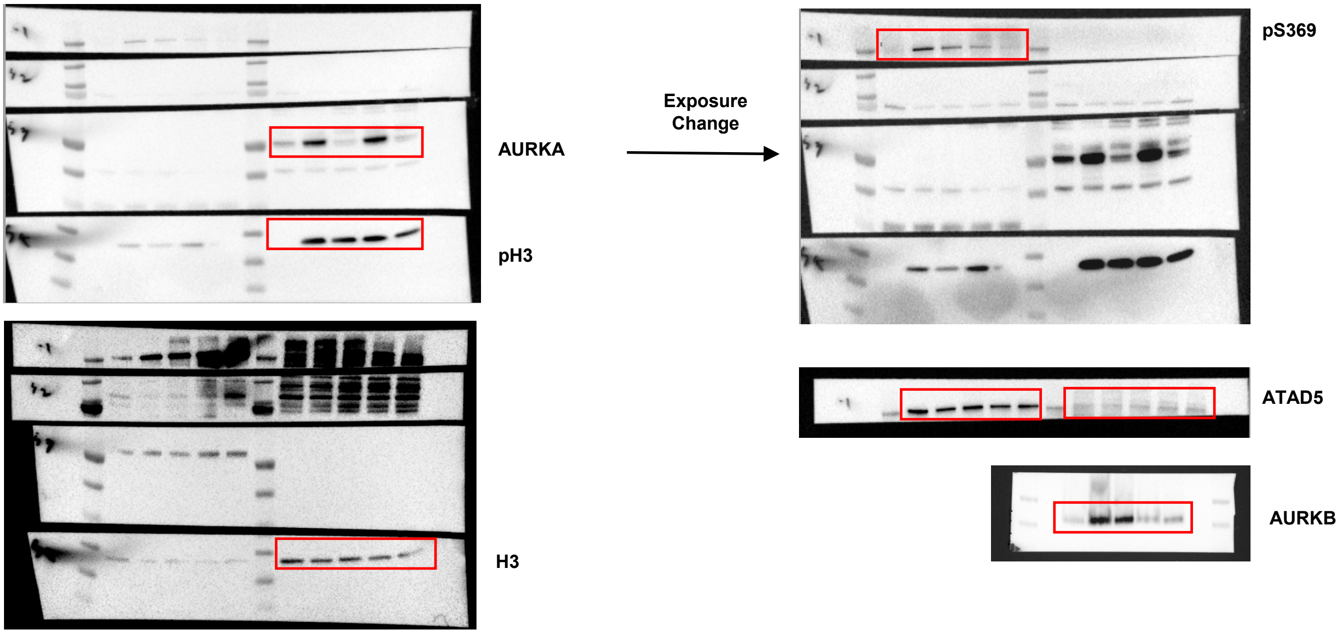

2I

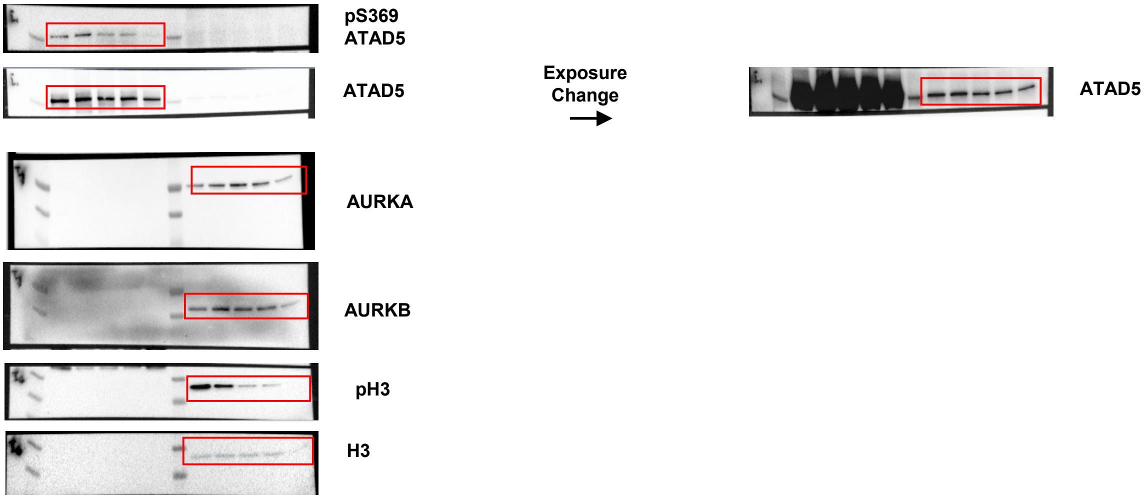

Supplementary Figure 5 (continued)

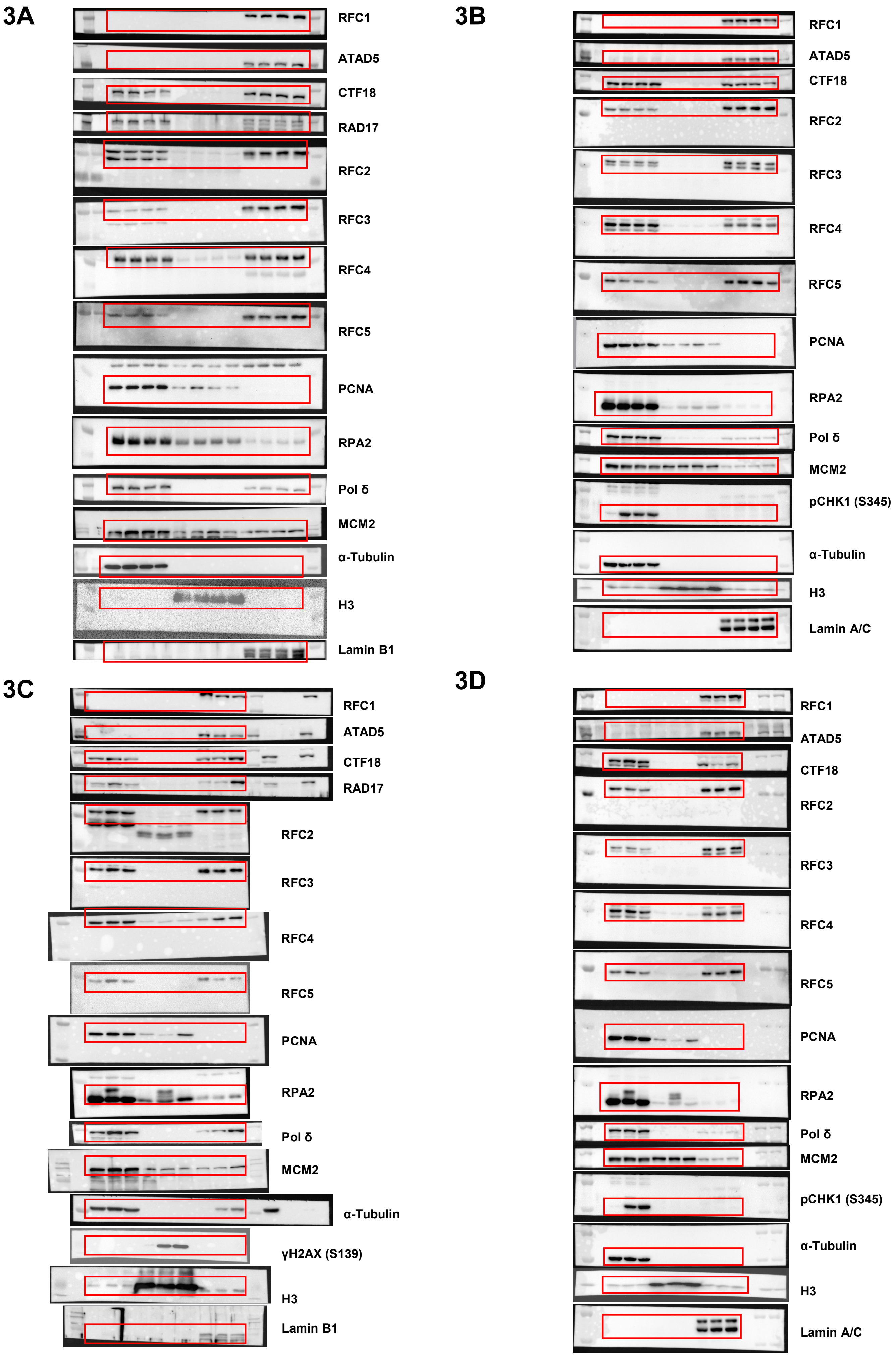

Supplementary Figure 5 (continued)

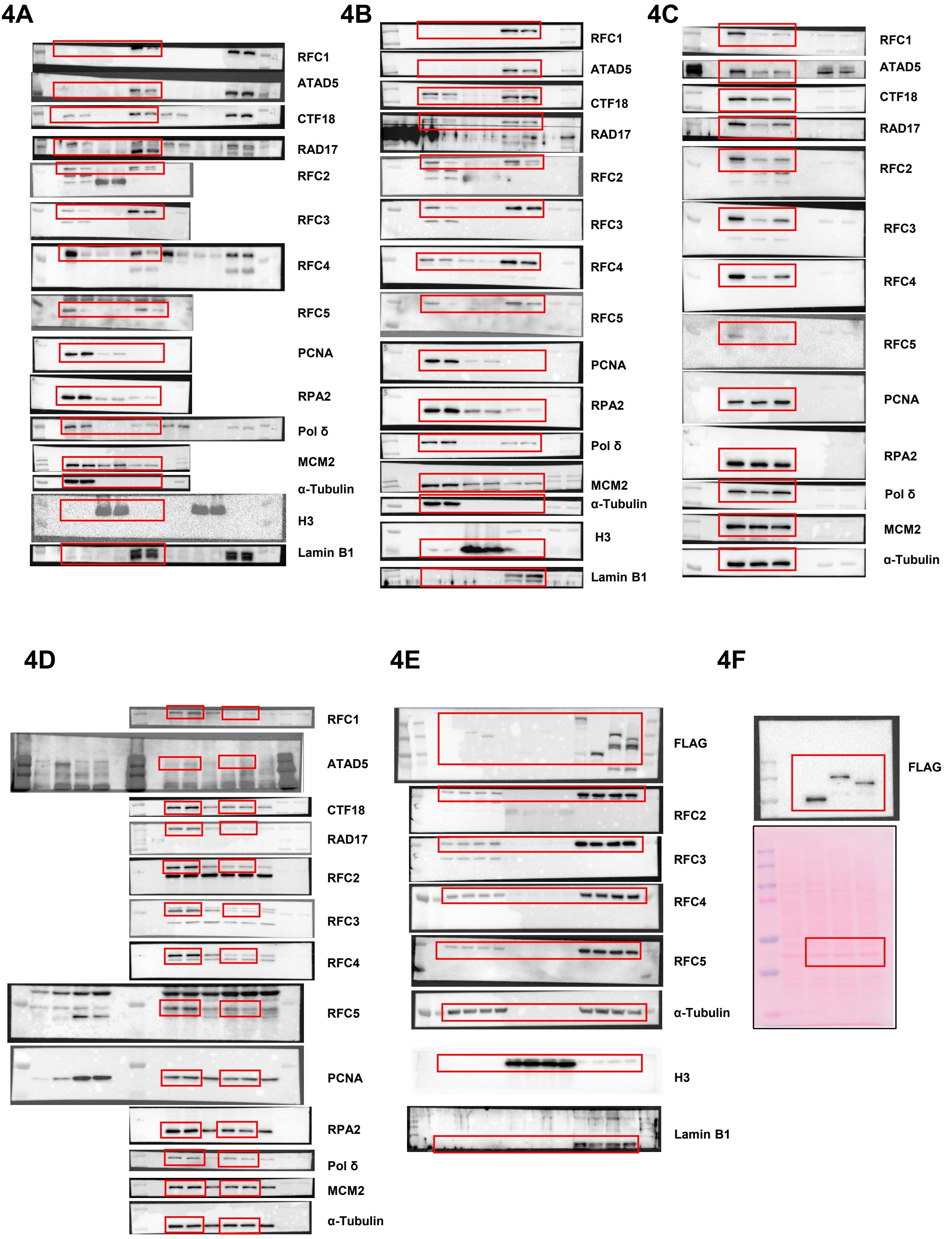

Supplementary Figure 5 (continued)

4G

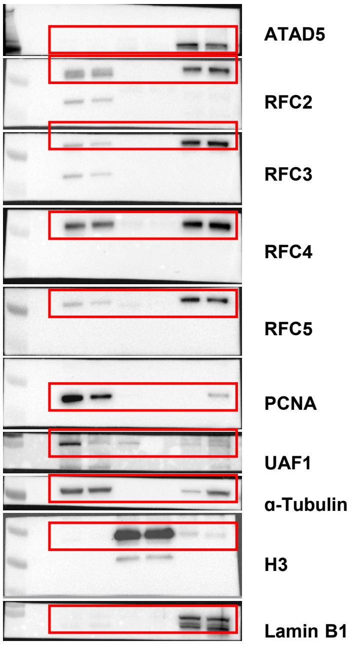

4H

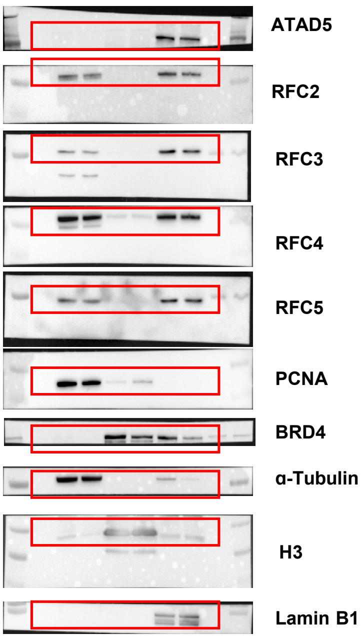

5B

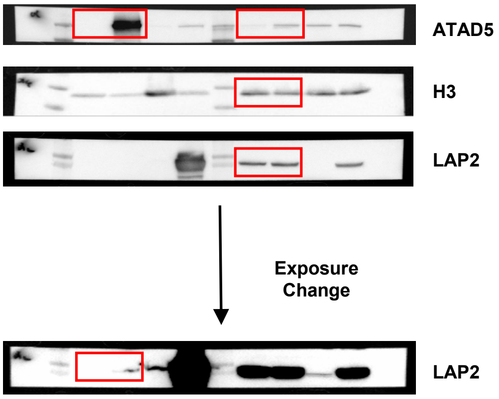

5C

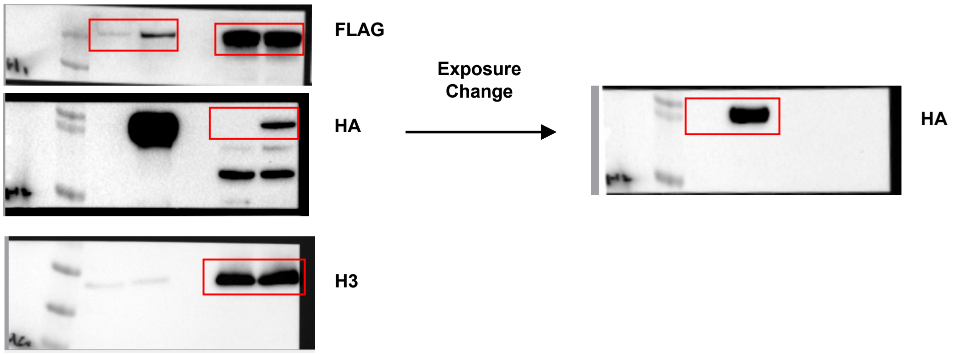

5D

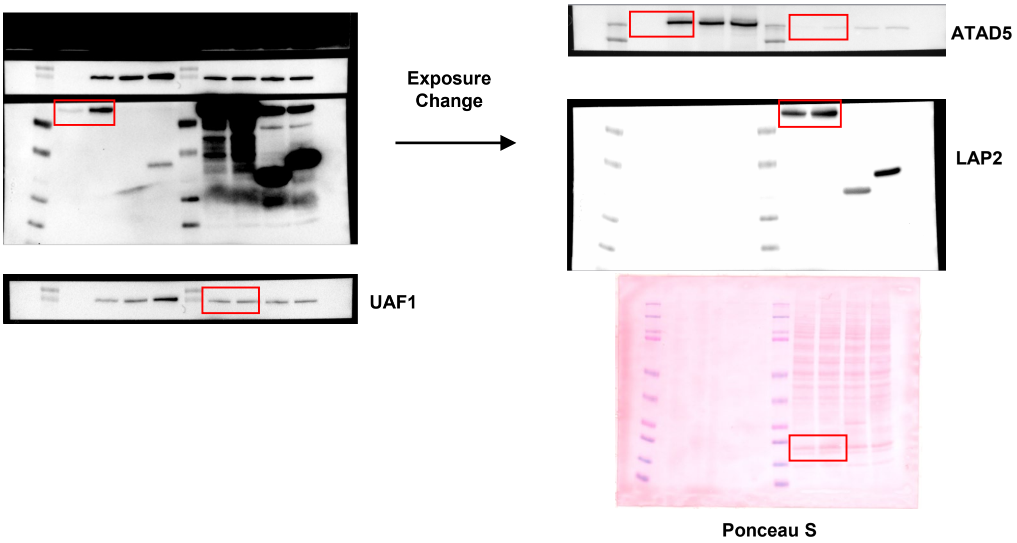

Supplementary Figure 5 (continued)

5E and 5F

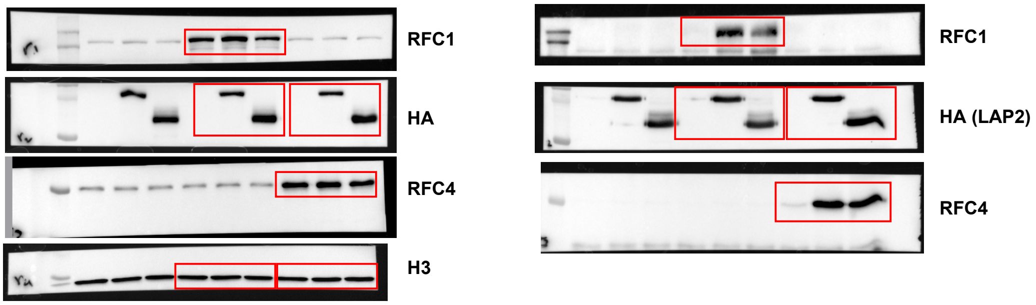

5G

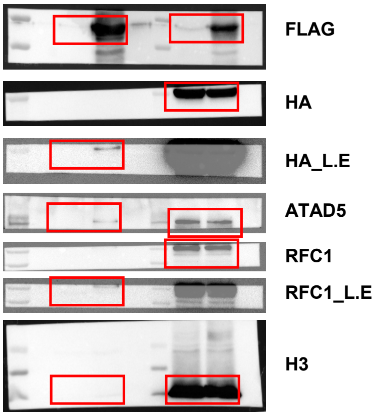

5H

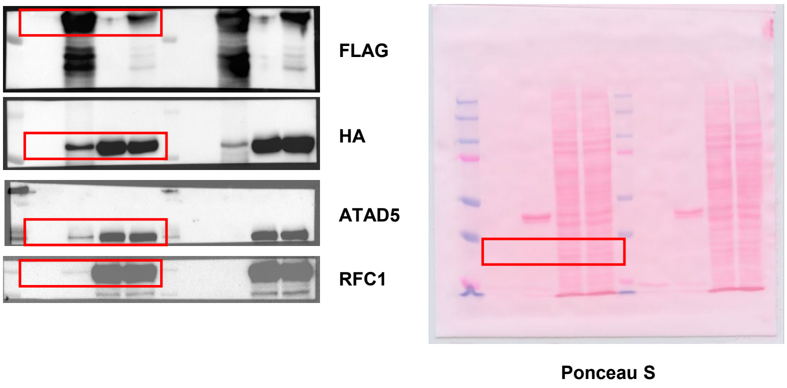

5I

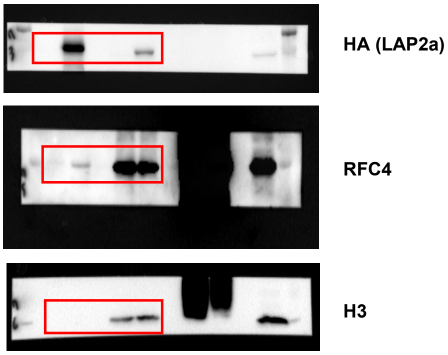

Supplementary Figure 5 (continued)

6A

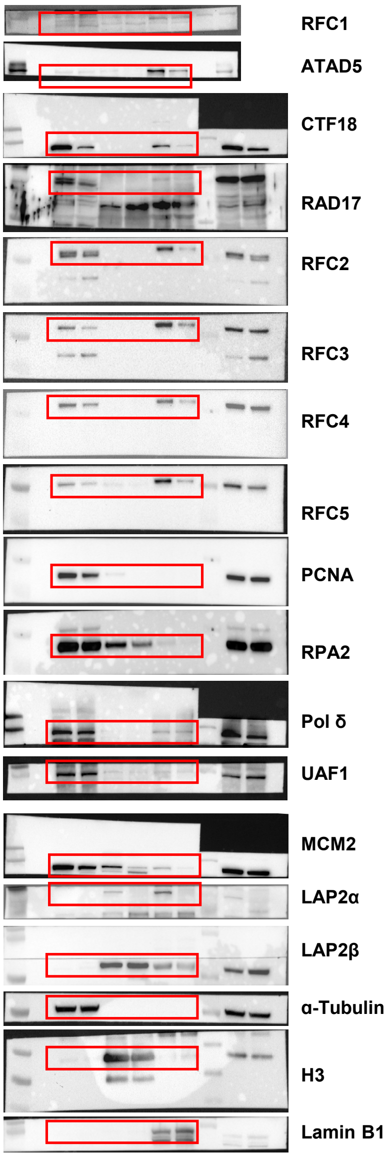

6B

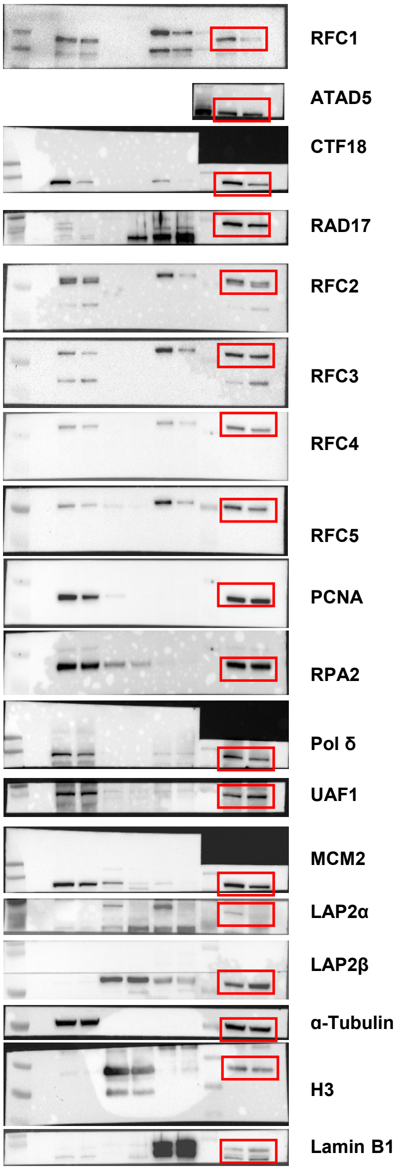

Supplementary Figure 5 (continued)

Supple. Figure 1

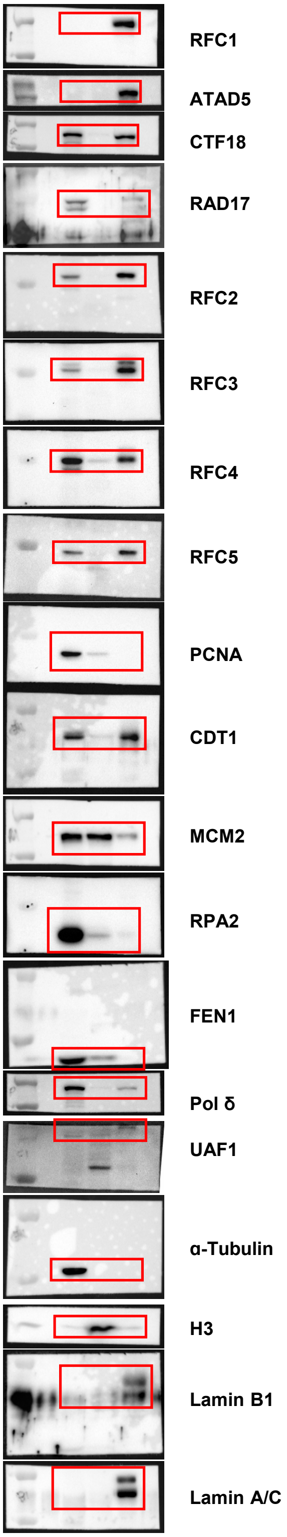

Supple. Figure 2

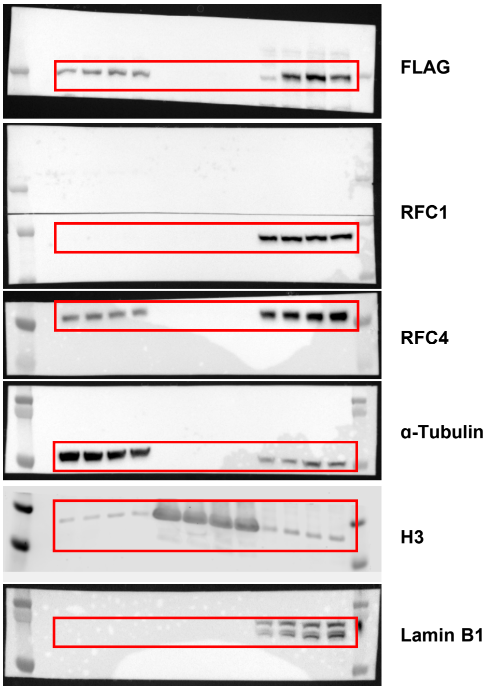

Supplementary Figure 5 (continued)

Supple. Figure 3A

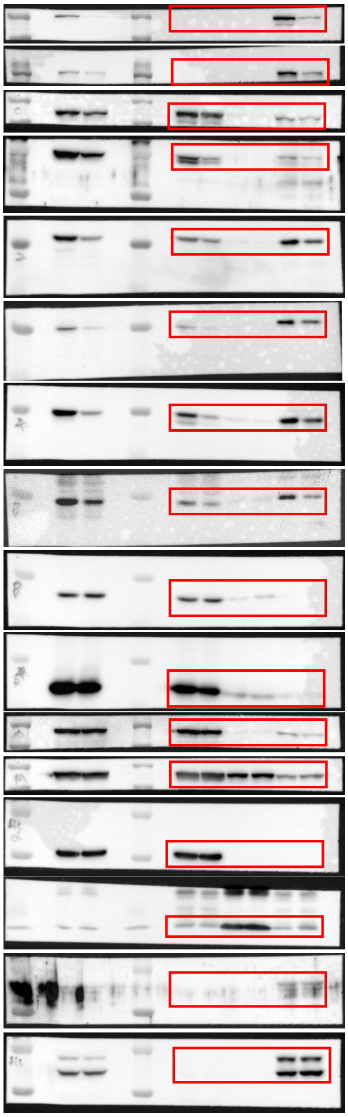

Supple. Figure 3B

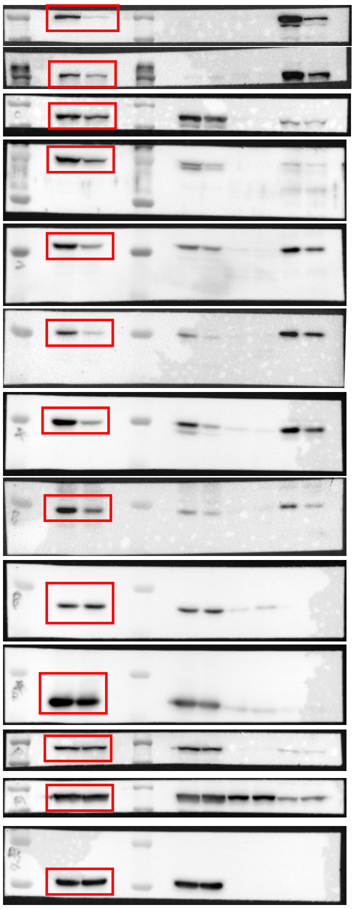

Supplementary Figure 5 (continued)

Supple. Figure 4A

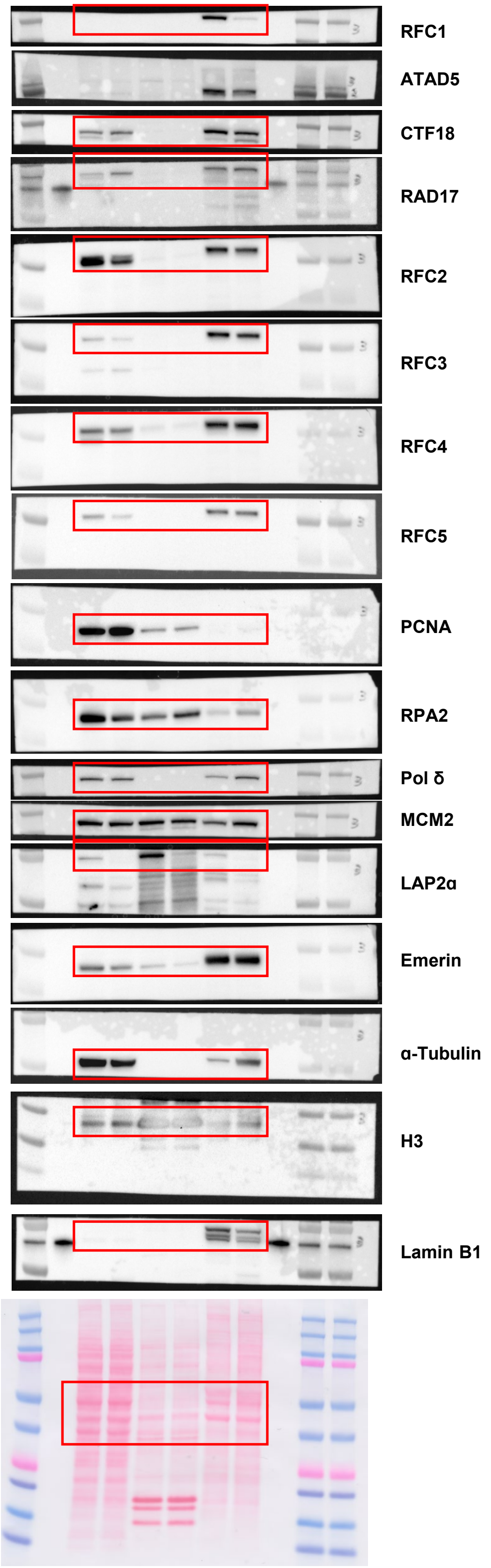

Supple. Figure 4B

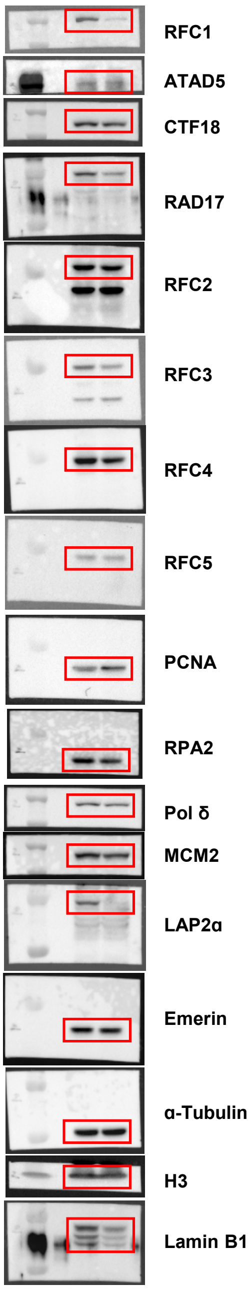

Supplementary Figure 5. Uncropped immunoblot images corresponding to Figure 1-6 and Supplementary Figure 1-4.
